# *Pomacea canaliculata* Ampullar Proteome: A Nematode-Based Bio-Pesticide Induces Changes in Metabolic and Stress-Related Pathways

**DOI:** 10.3390/biology10101049

**Published:** 2021-10-15

**Authors:** Federica Boraldi, Francesco Demetrio Lofaro, Giulia Bergamini, Agnese Ferrari, Davide Malagoli

**Affiliations:** 1 Department of Life Sciences, University of Modena and Reggio Emilia, 41125 Modena, Italy; francescodemetrio.lofaro@unimore.it (F.D.L.); agnese.ferrari@unimore.it (A.F.); 2 Department of Chemical and Geological Sciences, University of Modena and Reggio Emilia, 41125 Modena, Italy; giulia.bergamini@unimore.it

**Keywords:** energy metabolism, gastropod, immunity, connective tissue, mollusc, rhogocyte, ultrastructure

## Abstract

**Simple Summary:**

*Pomacea canaliculata* is a South American invasive freshwater snail, affecting biodiversity, crop production and public health, now retrieved in Asian, North American and European countries. The identification of molecules connected to *P. canaliculata* adaptability may prove helpful in developing strategies that could overcome the snail’s resilience and stop its spread in non-original countries. This research presents the changes occurring in the proteome of a small organ, i.e., the ampulla, after challenging the snails with a nematode-based bio-pesticide. Transmission electron microscopy (TEM) analysis demonstrated that this organ has a complex connective ultrastructure and contains rhogocytes. TEM also confirmed the ampulla as a deposit of nitrogen-based material. After exposure to the nematode-based bio-pesticide, significant changes were observed for enzymes mainly involved in antioxidant defence, energy metabolism and cytoskeletal dynamics. These changes recall the systemic oxidative stress response that the snails undergo during the arousal after aestivation/hibernation, a physiological response involving other organs that, similarly to the ampulla, store nitrogen-based compounds. As fundamental players of the response against bio-pesticides and environmental cues, the enzymes identified in this research and involved in stress-related pathways may represent a suitable target for the efficacious and sustainable control of *P. canaliculata* spread.

**Abstract:**

*Pomacea canaliculata* is a freshwater gastropod known for being both a highly invasive species and one of the possible intermediate hosts of the mammalian parasite *Angiostrongylus cantonensis*. With the aim of providing new information concerning *P. canaliculata* biology and adaptability, the first proteome of the ampulla, i.e., a small organ associated with the circulatory system and known as a reservoir of nitrogen-containing compounds, was obtained. The ampullar proteome was derived from ampullae of control snails or after exposure to a nematode-based molluscicide, known for killing snails in a dose- and temperature-dependent fashion. Proteome analysis revealed that the composition of connective ampulla walls, cell metabolism and oxidative stress response were affected by the bio-pesticide. Ultrastructural investigations have highlighted the presence of rhogocytes within the ampullar walls, as it has been reported for other organs containing nitrogen storage tissue. Collected data suggested that the ampulla may belong to a network of organs involved in controlling and facing oxidative stress in different situations. The response against the nematode-based molluscicide recalled the response set up during early arousal after aestivation and hibernation, thus encouraging the hypothesis that metabolic pathways and antioxidant defences promoting amphibiousness could also prove useful in facing other challenges stimulating an oxidative stress response, e.g., immune challenges or biocide exposure. Targeting the oxidative stress resistance of *P. canaliculata* may prove helpful for increasing its susceptibility to bio-pesticides and may help the sustainable control of this pest’s diffusion.

## 1. Introduction

The in-depth understanding of the biological features of invasive species and of the ecological interactions between parasite reservoirs and humans is necessary for predicting and controlling human exposure and environmental risks [1,2]. Molluscs include species of economic value but also invasive pests, and studies on their stress response can give a significant contribution to parasitology, conservation biology and human welfare, among others [3]. Freshwater gastropods include recognised models for translational science such as *Lymnaea stagnalis* [4], but also include invasive species and parasite-bearing vectors, e.g., *Biomphalaria glabrata* [3] and *Pomacea canaliculata* [5]. *P. canaliculata* belongs to a diversified genus [6] and it is a highly invasive species, with relevant reproductive and fast growth rates [7,8]. Numerous approaches have been adopted for controlling *P. canaliculata* spread [9,10], and recently we have observed the efficacy of a nematode-based molluscicide [11]. The nematodes contained in the biocide promoted a significant immune response in different organs on the basis of the temperature and the dose utilised. How the parasitic nematode, *Phasmarhabditis hermaphrodita*, contained in the molluscicide can overcome the immune system of *P. canaliculata* and other molluscs is not well-defined [11,12,13,14], but collected data suggested that the immune response raised by infected *P. canaliculata* may be systemic [11], and could involve cellular and humoral components. Studies on the immune system of *P. canaliculata* are now available [11,15,16,17,18,19,20,21,22], together with information about their genome [23], organ specific transcriptomes [24,25] and proteomes [17,26,27]. The interest towards this model is due to its invasiveness [28], neurotoxicity [29], human parasite hosting [30] and observations about its capability to regenerate [31,32,33,34]. The systemic immune response in this snail involves circulating and tissue-resident haemocytes, together with different organs [11,16,18,19,20,21,31,35,36]. The ampulla has been described as a capacious expansion of the anterior aorta, acting as a potential compensation chamber for the haemolymph when the animal is forced to retract into its shell [34]. Among other functions, a potential role for amoebocyte proliferation was also proposed, but no evidence for haemocyte mitosis was observed in successive experiments [37]. The hypothesis that the ampulla might serve as a haemocytic reservoir [14] remained unproven. Biochemical and ultrastructural evidence also suggested the involvement of the ampulla, together with other organs, in the anti-oxidant stress response associated with the aestivation/hibernation processes [38,39]. In these respects, the oxidative stress observed in the apple snail mimics to some extent the oxidative burst that is associated with ischemia–reperfusion injury [40], and this capacity of resisting a systemic oxidative burst could also represent a resource in case of other stressors, e.g., exposure to a biocide or an immune challenge. In this article, we present the analysis of the ampulla’s proteome variations after exposing snails to a nematode-based molluscicide. Together with details on ampulla ultrastructure, our experiments revealed that *P. canaliculata* responded to the *P. hermaphrodita*-based bio-pesticide with a rapid change of ampulla proteome, modifying the expression levels of proteins related to oxidative stress response and energy metabolism.

## 2. Materials and Methods

### 2.1. Snail Maintenance and Treatment

*P. canaliculata* specimens were imported in 2012 from Trans Aquarium Fish SrL (Scalenghe, Italy) and then bred in the aquarium facility of the Department of Life Science (University of Modena and Reggio Emilia, Modena, Italy). The snails were maintained in aerated tap water at 25 ± 1 °C, dark/light cycle of 14/10 h. Approximately 90% of the water contained in each tank was replaced twice a week and, immediately after water change, animals were fed ad libitum with mixed types of green salad, suitable for human consumption. Eighteen adult snails (shell diameter between 35 and 50 mm) were used in the experimental protocols (9 control and 9 Nemaslug^®^-exposed snails). Exposure to 1.7 g/L Nemaslug^®^ (BASF SE, Ludwigshafen, Germany), a commercially available molluscicide based on the nematode *Phasmarhabditis hermaphrodita*, was performed for 24 h at 25 ± 1 °C, according to Montanari et al. [11]. When used at these concentrations and temperatures, Nemaslug^®^ kills approximately 14% of snails within one week. Control and Nemaslug^®^-exposed snails were anaesthetised in ice for 20 min before ampulla dissection under a dissection microscope.

### 2.2. Transmission Electron Microscopy (TEM)

The information concerning ampulla ultrastructure in control snails was necessary to correlate it with the proteome analysis. For ultrastructural analysis, small pieces of ampulla were processed as already described [41]. Briefly, samples were fixed in 2.5% glutaraldehyde in 0.1 M cacodylate buffer and then post-fixed in 1% OsO_4_ in the same buffer. Pieces were dehydrated and embedded in Epon 812 resin. Semi-thin sections were cut at 0.25 μm and stained with 1% toluidine blue. Ultrathin sections (60 nm) were cut with a diamond knife and mounted on a copper grid and unstained samples were examined using a Talos F200S G2 transmission electron microscope (Thermo Fisher Inc., Fisher Scientific Italia, Rodano Italy) performed at 200 kV. Energy dispersive spectroscopy analysis was performed on different areas of samples. The selected areas’ electron diffraction pattern was used to analyse the possible crystalline nature of the spheroidal bodies.

### 2.3. Protein Extraction and Preparation

For each experimental condition, we analysed 3 biological replicates, each obtained by pooling 3 ampullae. Frozen ampullae were homogenised in RapiGest^TM^ SF (Waters SpA, Milan, Italy) 0.2% using a G19 needle and subsequently incubated 30 min in ice. Samples were further incubated 5 min at 100 °C and centrifuged at 9500g for 5 min to collect the supernatant. Protein concentration was evaluated by the Bradford method [42]. For each replicate, in solution digestion was performed using 50 µg of proteins. Briefly, proteins were reduced and alkylated by 5mM dithioerythritol and 15 mM iodoacetamide, respectively. Proteins were digested with trypsin (Promega, Madison, WI, USA) solution buffered in 50 mM NH_4_HCO_3_ overnight at 37 °C using an enzyme to protein ratio of 1:50 (*w*/*w*). RapiGest^TM^ SF (Waters) was removed using 0.5% formic acid and centrifuged at 10000g for 10 min at RT. Tryptic peptides were collected from supernatant and were ready-to-use for LC-MS/MS analysis. All the reagents were purchased from Sigma-Aldrich (Merk KGaA, Darmstadt, Germany) unless otherwise stated.

### 2.4. LC-MS/MS

An UHPLC ultimate 3000 system coupled online to a Q Exactive Hybrid Quadrupole-Orbitrap Mass Spectrometer (Thermo Fisher Scientific, Waltham, MA, USA) was used, as already described [43] with some modifications. Chromatographic separation of peptides took place in a reverse-phase C18 column (2.1 μm ID × 50 mm, 1.8 μm, Zorbax, Merk KGaA, Darmstadt, Germany) and elution was performed using a binary system of solvent. Mobile phase A consisted of 0.1% formic acid in ultrapure water. Mobile phase B was 0.1% formic acid in acetonitrile. For separation, a linear binary gradient was applied: 2–3% B in 5 min, to 28% B in the next 59 min, and then 90% B in 7 min. The column was maintained at 30 °C and the flow rate employed was 0.3 mL/min. Precursor ion detection and fragment ion detection were performed in an m/z-range from 200 to 2000. Data acquisition was controlled by Xcalibur 2.0.7 Software (Thermo Fisher Scientific, USA).

### 2.5. Data Analysis

Raw ms/ms data were converted by msConvert ProteoWizard (v.3.0.19239) in MGF file using default settings and uploaded to the MASCOT server (v.2.7.0) for MS/MS Ion Search. Search was performed using the ASM307304v1 database [23] and Uniprot (2018_05) restricted to Protostomia. Furthermore, parameters for identification included: (i) trypsin as enzyme with 1 of maximum missed cleavage; (ii) mass error tolerances for precursor and fragment ions set to 10 ppm and 0.02 Da, respectively; (iii) peptide charge (2+, 3+, 4+); (iv) protein mass no restriction; and (v) carbamidomethyl cysteine (C) set as fixed modification while deamidation of asparagine and glutamine (NQ) and oxidation of methionine (M) were considered as dynamic modification. Only confident peptides identified with a false discovery rate ≤1 and proteins with at least two unique peptides were exported. Proteins identified only in one sample per condition were excluded by further analysis. We performed label-free quantification using a tandem mass spectra counting approach [44], as already described [45,46]. Briefly, the exponentially modified protein abundance index (emPAI), integrated into Mascot, was calculated as the ratio between the number of experimentally observed peptides per protein and the number of theoretically observable peptides per protein. All emPAI values obtained were normalised, dividing each emPAI value for the sum of all emPAI values [47]. The expression levels of the proteins identified in ampullae isolated from control (AmpC) and Nemaslug^®^-exposed snails (AmpN) were evaluated by fold changes. For each protein, fold change was obtained by dividing the emPAI average of AmpN by that of AmpC. The fold change was transformed using the log_2_ function and the *p*-value, calculated by Student’s *t*-tests, was transformed using the -log_10_ function. Proteins were considered up- or down-regulated when the log_2_ fold change was more than 1 and less than −1, respectively, and the *p*-value < 0.05.

### 2.6. Bioinformatic Analysis

GO enrichment analysis was performed following Blast2GO (v. 5.2.5). Firstly, search homology parameters, NCBI pBLAST, include: (i) nr Blast DB; (ii) Eukaryota Taxonomy filter; (iii) Blast e-value 1,0E-3; (iv) 20 numbers of hits; (v) Blast descriptor annotator; (vi) low complexity filter. The pBLAST output was mapped and annotated with default settings. Lastly, EMBL-EBI InterPro was carried out with default settings, and annotations were merged with those of BLAST. In order to identify functional regions (domain) of ECM proteins, a biosequence analysis was performed using the profile hidden Markov model of HMMER (v.3.3.1, http://hmmer.org/; accessed on 16 February 2021) software integrating profile databases such as Pfam. Heat map analysis of protein expression levels was performed using R-software (v.4.0.5) and scaled in row direction (Z-score). Heat maps reorder both variables and observations using a clustering algorithm that computes the distance between each pair of rows and columns and orders them by similarity.

## 3. Results

### 3.1. Ultrastructure Analysis Revealed a Variety of Cells in Ampullae

**Figure 1:**
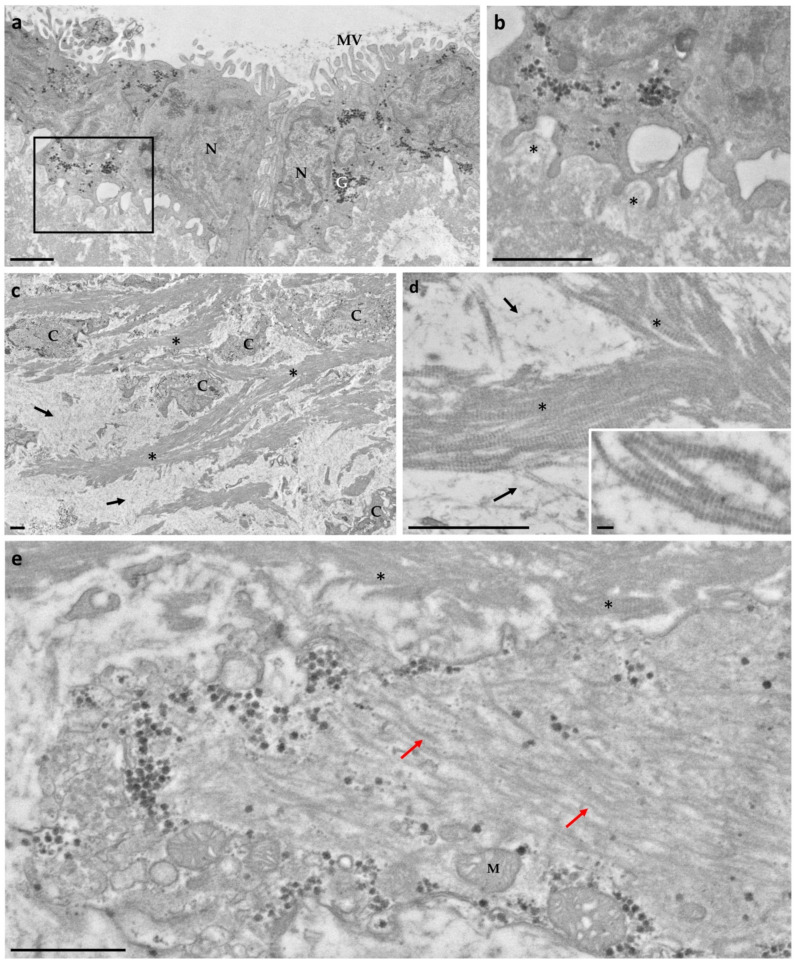
Electron microscopy of the ampulla obtained from *P. canaliculata*. (**a**) The external surface was covered by a monolayer of epithelial cells with numerous microvilli (MV). (**b**) Cell membrane protrusions connected the epithelial cells to the basement membrane (*). (**c**) Numerous cells (C) appeared to be embedded in the extracellular matrix, which consisted of collagen fibrils (*) spread or assembled into bundles. (**d**) Fibrillar structures observed in connective tissue: thin fibrils (arrows) and longitudinally sectioned collagen fibrils showing the typical banding (*). Insert: collagen fibrils at higher magnification. Scale bar = 100 nm. (**e**) Representative image of cell with abundant cytoplasmic filaments (red arrows). Mitochondria (M), glycogen granules and vesicles were arranged closely to the plasma membrane. Scale bar = 1 μm. G = glycogen; N = nucleus.

The ampulla was outlined by a single layer of cells presenting a large nucleus and glycogen aggregates (Figure 1a). Several microvilli were present on the apical membrane, facing the pericardial cavity, whereas, on the basal side, cell membrane protrusions connected epithelial cells to a basement membrane (Figure 1b, asterisks).

Below the basement membrane, the extracellular matrix (ECM) of the underlying connective tissue contained numerous fibrillar structures. Some of these fibrils exhibited a typical collagen binding and were mainly assembled into either parallel bundles or branched structures (Figure 1c,d). Other fibrils were thin and spread into the matrix (Figure 1d), possibly mediating the interactions between fibrillar structures and other matrix macromolecules.

Within the connective tissue, numerous cells, morphologically heterogeneous for dimension and shape, were present. A number of cells of irregular shape exhibited a cytoplasm filled with filaments (Figure 1e, red arrows). At the periphery of the cells, numerous large mitochondria, glycogen granules and small vesicles were present (Figure 1e).

**Figure 2:**
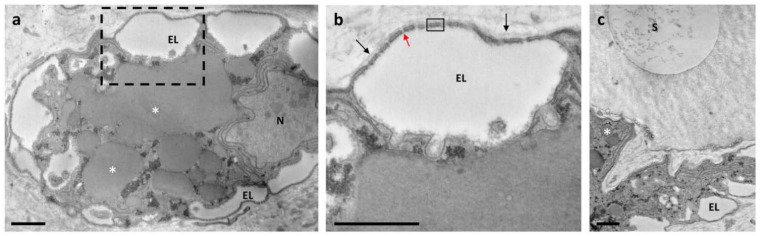
Electron microscopy of rhogocytes found in connective tissue of ampulla. (**a**) Rhogocytes with large nucleus (N), several big electron-dense granules (asterisk), abundant endoplasmic reticulum and extracellular lacunae (EL). (**b**) Magnification of the area marked in (**a**) showing EL, cytoplasmic bar (rectangle), diaphragmatic slits (red arrow) and extracellular lamina (black arrows). (**c**) Rhogocyte close to a cell with cytoplasmic spheres (S). Scale bar = 1 μm.

Rhogocytes, also known as pore cells or Leydig cells [48,49], could be recognised within the connective tissue (Figure 2a–c). A large nucleus, numerous dense granules, a prominently rough endoplasmic reticulum and slit complexes, located at the cell periphery, were typical features of these cells [48,49]. Moreover, rhogocytes exhibited a typical invagination of the plasma membrane forming subsurface cisternae [50] or extracellular lacunae [49], which were linked via cytoplasmic bars connected by a thin diaphragm (diaphragmatic slits) (Figure 2a–c). Extracellular lacunae were heterogeneous in size and usually empty, although granular material could be occasionally observed. Some vesicles filled with particles were close or fused with extracellular lacunae (Figure 2a,b). Rhogocytes were surrounded by an extracellular lamina and were observed either as single cells or organised in small clusters, and sometimes they were in proximity to cells containing cytoplasmic spheres (Figure 2c).

**Figure 3:**
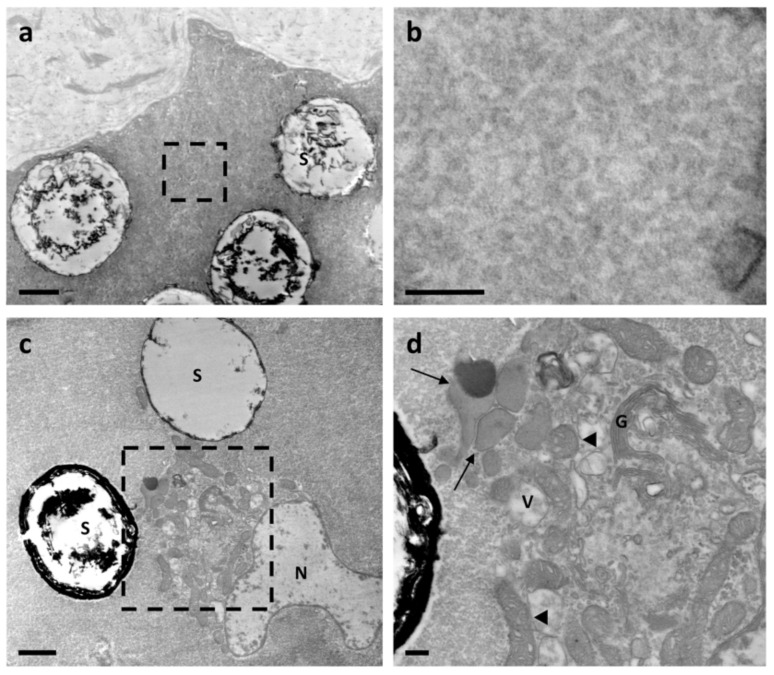
Electron micrography of cells with cytoplasmic spheres. (**a**) Some mitochondria (arrowheads) were located close to plasma membrane of a cell with cytoplasm full of electron-dense clumps. Insert is magnification of a mitochondrion. (**b**) Magnification of the area marked in (**a**) showing electron-dense clumps filling the cell cytoplasm. Bar = 100 nm. (**c**) Mitochondria (arrowheads), nucleus (N) and some vacuoles were visible at peripheral cytoplasmic area. (**d**,**e**) Cytoplasmic spheres that are empty or filled with electron-dense material. Scale bar = 1 μm if not differently specified.

**Figure 4:**
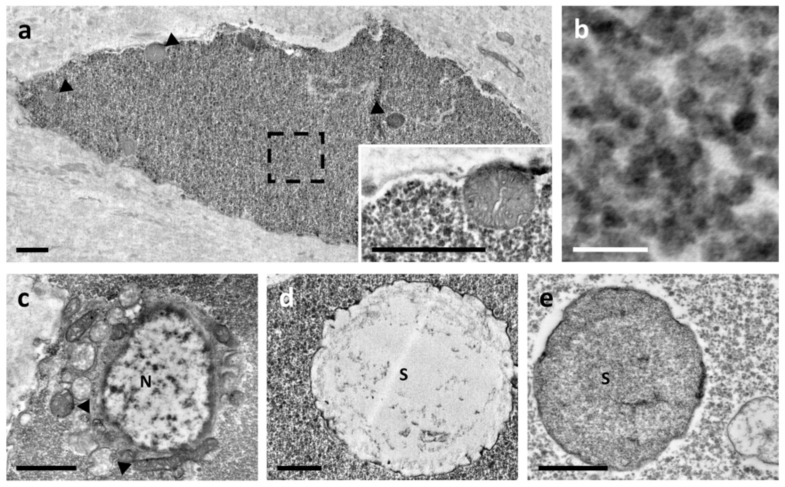
Electron micrography of cells with cytoplasmic spheres. (**a**) Cell with some spheres embedded in cytoplasm. Scale bar = 1 μm. (**b**) Magnification of the zone marked in (a) showing that the cytoplasm was full of globular structures of low electron density. Scale bar = 100 nm. (**c**,**d**) Nucleus (N), mitochondria (arrowheads), Golgi apparatus (G), electron-dense granules (arrows) and some vacuoles (V) were visible close to cell plasma membrane. Scale bar = 1 μm and the enlargement scale bar = 250 nm.

**Figure 5:**
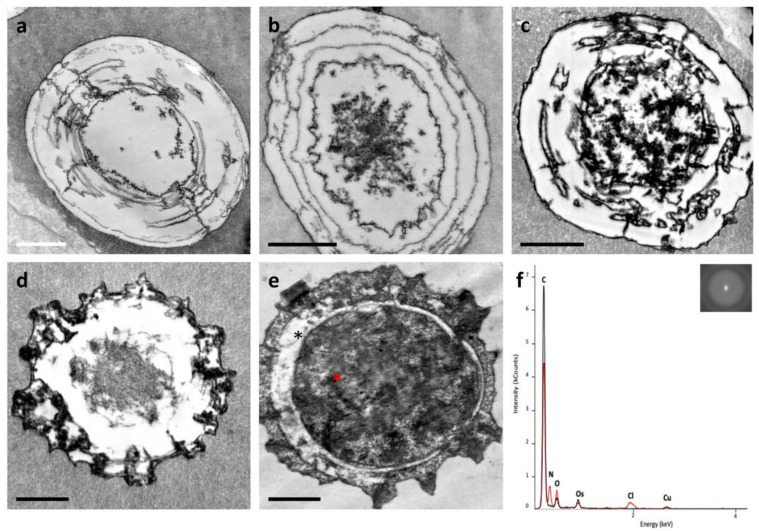
Electron micrography of spheres observed in cells. (**a**–**c**) Different spheres with regular and smooth contours. (**d**,**e**) Spheres with contours like a “wagon’s wheel”. (**f**) Energy dispersive spectroscopy (EDS) spectra derived from X-ray microanalysis. The black and red lines were obtained from areas marked with black and red asterisks in (**e**), respectively. Both EDS spectra displayed the peaks of O, C, Os, Cl and Cu, of which the last three derived from the sample preparation. The red line also showed the presence of N. On the top right are selected area electron diffraction patterns obtained in the area marked with a red asterisk in (**e**). Scale bar = 1 μm.

The cells with cytoplasmic spheres (Figure 3, Figure 4 and Figure 5) were on the luminal side of the ampulla and presented an abundant cytoplasm full of electron-dense material and peripheral mitochondria (Figure 3a,b). Their nuclei were small, adjacent to the cell membrane, surrounded by mitochondria with dilated cristae and small vacuoles (Figure 3c) and contained mainly euchromatin with small heterochromatin clumps (Figure 3c). Cytoplasmic spheres appeared either empty or filled with electron-dense material, which in its turn could be arranged in clumps or in fibrillar structures (Figure 3d,e). Cells exhibiting a prevalence of electron-dense spheres were also characterised by small vesicles close to the plasma membrane (Figure 4a) and by a cytoplasm with poorly electron-dense globular structures (Figure 4b). Golgi apparatus, elongated mitochondria with dilated cristae and electron dense granules were also present (Figure 4c,d).

The electron-dense cytoplasmic spheres had an approximate diameter of 2–4 µm. They consisted of either fine microgranular material, radially dispersed, and concentric electron-dense rings (Figure 5a–c) or a morphology similar to a “wagon’s wheel” [51] (Figure 5d,e). The selected area diffraction patterns indicated that the electron-dense material did not contain crystalline species (Figure 5f). X-ray microanalyses, performed on low and high electron density material, revealed peaks of C, O, Cu, Cl and Os, of which the last three elements were artefacts due to the copper grid and to the solutions used for the embedding phase. The peak of N was only found in dense electron material (Figure 5f), indicating an unequal distribution of this element within the spheres.

### 3.2. Protein Profile of Control Ampulla

**Figure 6:**
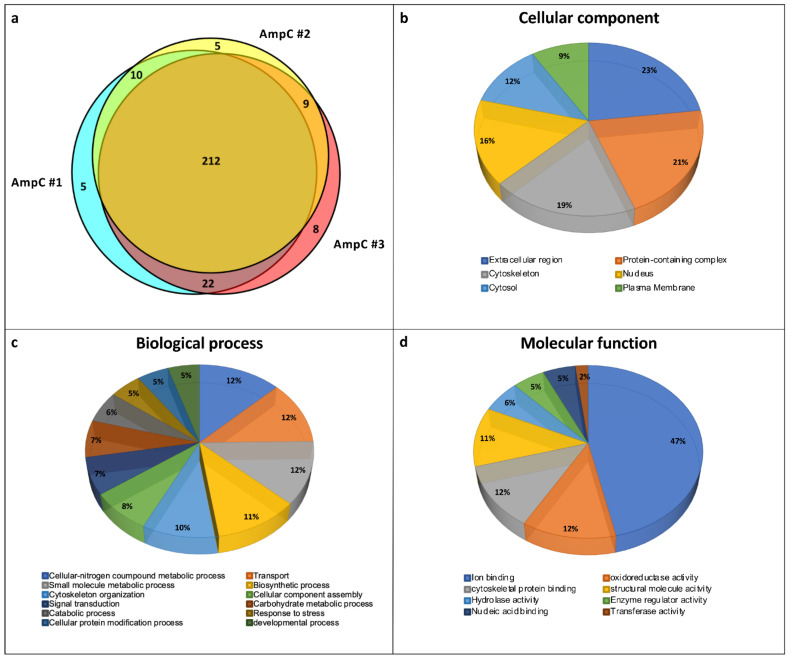
Proteomic analysis. (**a**) Venn diagram of total proteins identified in each of three biological replicates (#1, #2 and #3). The proteins identified in at least two biological replicates (349) were used for further analysis. (**b**–**d**) Gene ontology analysis related to cellular component, biological process and molecular function, respectively. The percentage of proteins enriched in each category is indicated.

The proteomic profile obtained from the control ampulla (AmpC) contained 271 proteins, of which 253 were found in at least two biological replicates and were considered as reliable identifications (Figure 6a and Table S1). These 253 proteins underwent an automated gene ontology (GO) analysis (Figure 6b–d), evidencing “Cellular Component”, extracellular region (23%), protein-containing complex (21%) and cytoskeleton (19%) as more frequent (Figure 6b). For “Biological Process”, proteins were mainly involved in cellular nitrogen compound metabolic processes (12%), transport (12%), small molecule metabolic processes (12%), biosynthetic processes (11%) and cytoskeleton organisation (10%) (Figure 6c). Finally, for “Molecular Function”, proteins were classified for their ion binding (47%) or oxidoreductase (12%) activity (Figure 6d).

**Figure 7:**
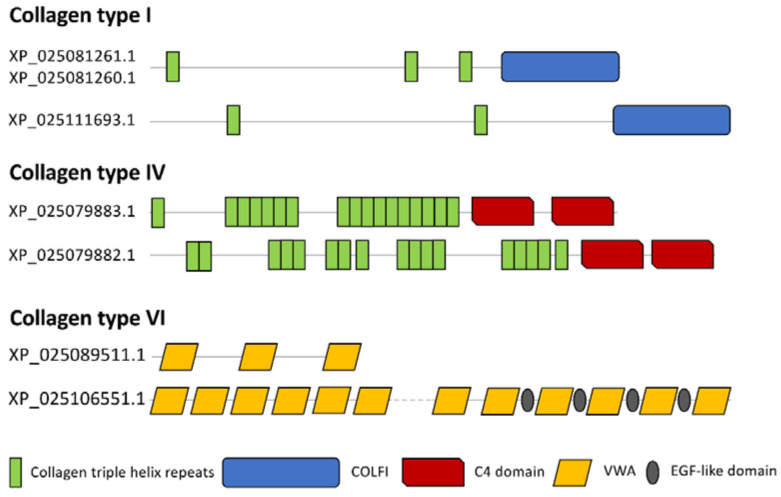
Schematic representation of domains identified in different collagen types.

Bearing in mind that our research organism could also present proteins without a corresponding GO term [52], we manually performed a further polypeptide classification, obtaining 33 and 220 proteins related to the ECM and to cellular components, respectively. Among the ECM we found several collagenous proteins: fibrillar-forming type I collagen (XP_025111693.1; XP_025081261.1; XP_025081260.1), network-forming type IV collagen (XP_025079883.1; XP_025079882.1) and microfibrillar type VI collagen (XP_025106551.1; XP_025089511.1) (Figure 7). Moreover, in collagen VI protein (XP_025106551.1), four EGF-like domains were interspersed between von Willebrand factor type A domains (VWA) (Figure 7).

**Figure 8:**
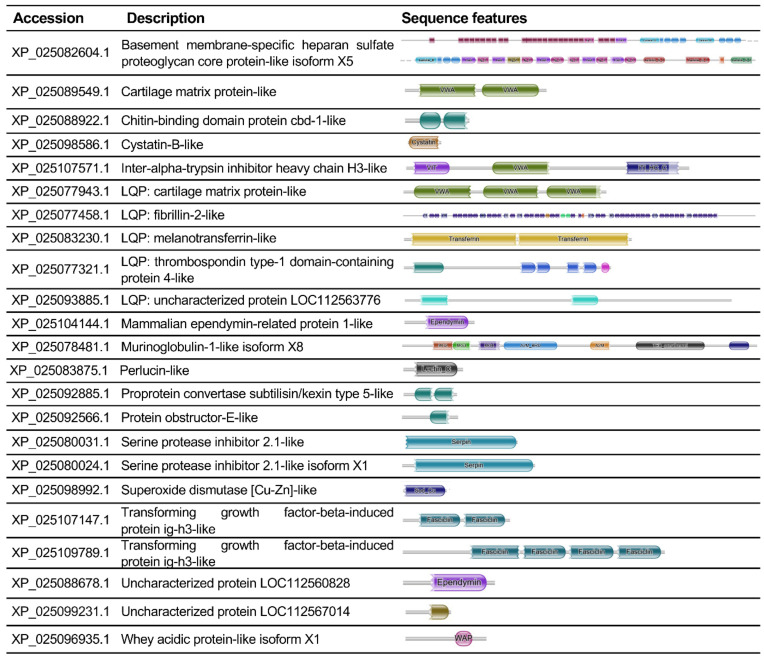
List of manually curated extracellular non-collagenous proteins. LQP = low-quality protein.

Among extracellular non-collagenous proteins (Figure 8), the presence of heparan sulphate/proteoglycan (XP_025082604.1), fibrillin (XP_025077458.1), ependymin-related proteins (XP_025104144.1; XP_025088678.1), members of the thrombospondin family (XP_025077321.1; XP_025089549.1) and two members of transforming growth factor-beta-induced protein ig-h3-like (TGFBI) (XP_025107147.1; XP_025109789.1), known to be involved in cell adhesion, migration and proliferation, was detected in ampullae [53]. Besides these structural proteins, we also found proteins involved in cell defence and physiology [54,55], such as a perlucin-like protein characterised by a lectin-C type domain (XP_025083875.1), cathepsin B (XP_025091465.1), cathepsin L1-like (XP_025097582.1), lysosomal aspartic protease (XP_025085667.1) and protease inhibitors such as cystatin (XP_025098586.1).

Among cellular proteins, we identified several polypeptides involved in cell contraction such as calponin-1-like (XP_025103230.1), myosin regulatory light chain LC-2 (XP_025095034.1 and XP_025095035.1), paramyosin-like (XP_025086366.1) and troponin (XP_025098601.1, XP_025085471.1 and XP_025086357.1) (Table 1). This suggested that cells containing cytoplasmic filaments (Figure 2a) may likely be muscle cells. The proteomic analysis also found several enzymes counteracting the potential negative effects of oxidative/nitrosative stress. Among the identified enzymes, Cu–Zn superoxide dismutase (SOD) (XP_025098992.1; XP_025107348.1) may be involved in the conversion from O_2_^•^^−^ to H_2_O_2_ [56] and catalase-like isoform X1 (XP_025098387.1) could further detoxify two H_2_O_2_ molecules into H_2_O and O_2_ [57]. Similarly, glutathione peroxidase-like (XP_025090868.1) protein can consume reduced glutathione for detoxifying H_2_O_2_ and also lipid peroxides generated by lipid peroxidation in *Schistosoma*-infected snails [57], while peroxiredoxin (XP_025109048.1) reduces H_2_O_2_, lipid hydroperoxides and ONOO^−^. Among the identified enzymes, there were several glutathione-S-transferases (XP_025113955.1; XP_025113405.1; XP_025107274.1), which are known to catalyse the nucleophilic attack of reduced glutathione on electrophilic centres of toxic compounds [56]. Finally, thioredoxin-like proteins (XP_025083634.1; XP_025092057.1 XP_025095496.1) can act as disulphide reductases or electron donors in the reduction of disulphide and dithiol. Control ampullae also contained haemocyanin G-type, units Oda to Odg-like (XP_025089564.1; XP_025089796.1), according to findings in other molluscs [58].

### 3.3. Nematode Exposure Determined a Rapid Change in the Protein Profile of Ampullae

**Figure 9:**
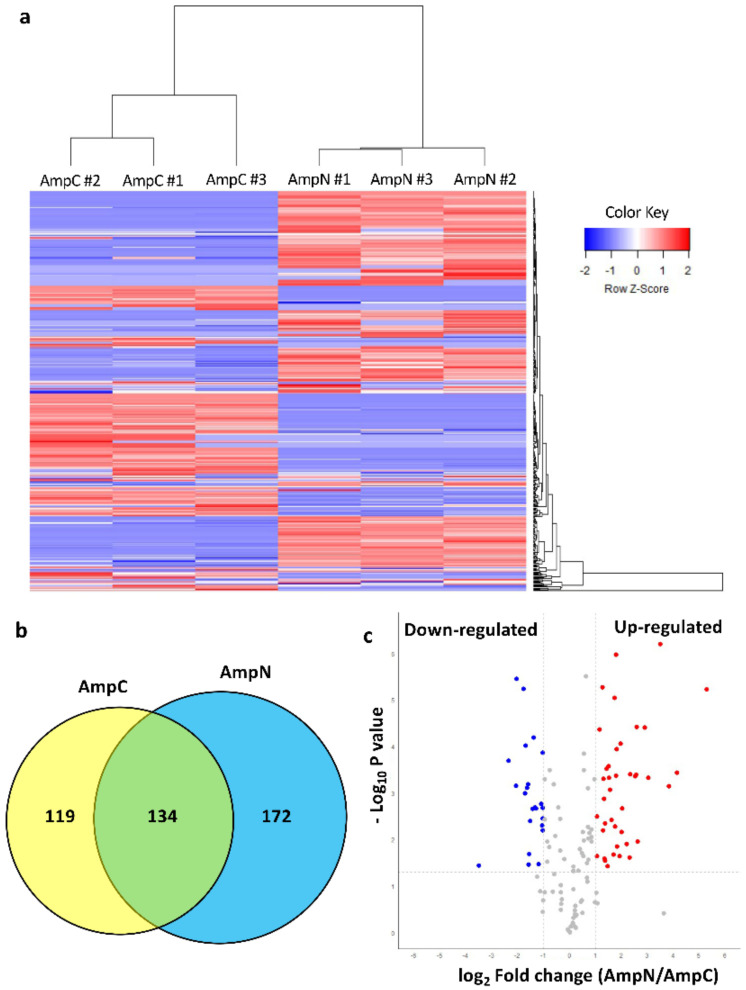
Heat map, Venn diagram and volcano plot showing protein expression in ampulla in absence (AmpC) or presence (AmpN) of nematode-based molluscicide. (**a**) Heat map of abundance of proteins identified in AmpC and in AmpN. The colour from blue to red represents the protein abundance level from low to high. (**b**) Venn diagram depicting unique and common proteins identified in AmpC and in AmpN. (**c**) Volcano plot displays the distribution of 134 common proteins with relative protein abundance (log_2_ fold change AmpN/AmpC) plotted against its significance levels (−log_10_ *p* value). Proteins with statistically significant differential expression (log_2_ fold change ≥ ±1 and *p* value < 0.05) are visualised in red and blue, indicating either the increased or the decreased polypeptides, respectively.

Recently, the nematode-based molluscicide, Nemaslug^®^, was tested on *P. canaliculata*, demonstrating dose-dependent effects especially on the anterior kidney and the gills [11]. Proteomic analysis of ampullae obtained from nematode-exposed *P. canaliculata* (AmpN) revealed 360 proteins, 265 common to all three biological replicates and 306 found in at least two biological replicates (Figure S1 and Table S1). The heat map shows the abundance of 253 and 306 polypeptides, which were found in at least two biological replicates of AmpC or AmpN, respectively (Figure 9a). The Venn diagram shows that 119 and 172 polypeptides were exclusively found in AmpC or AmpN, respectively, while 134 proteins were found in both experimental conditions (Figure 9b, Tables S2 and S3). A label-free quantitative proteomic analysis was applied to reveal differentially expressed common proteins (Table S3). Out of the 134 common proteins, 23 and 40 were significantly down- and up-regulated after nematode exposure, respectively (Figure 9c and Table 2).

### 3.4. Nematode Exposure Induced an Up-Regulation of the Antioxidant Defence

Upon nematode exposure, in the ampulla of *P. canaliculata*, the protein levels of glutathione S-transferase (XP_025113955.1; XP_025113405.1; XP_025107274.1), catalase-like (XP_025098387.1) and peroxiredoxin-2 (XP_ XP_025097674.1) were up-regulated (Table 2). These enzymes play a crucial role in detoxification and are important in determining the sensitivity of cells to external stress [59]. The increase in these enzymes could be interpreted as an adaptation mechanism of cells to nematode favouring, and therefore, snail survival in a stressful condition.

### 3.5. Nematode Exposure Induced a Stress Response

It is well known that cells respond to stressors (e.g., cold, UV and infection) by the over-expression of heat shock proteins (HSPs). Usually, HSPs are constitutively expressed in the cells and may have a rapid turnover, especially during stress exposures. HSPs are classified on the basis of their molecular weight and play a role in protein folding, assembly, degradation, remodelling and in localising proteins in an appropriated cellular compartment [60]. A significant increase in expression levels of several HSPs (i.e., HSP60 (XP_025095794.1); HSP70 (XP_025099490.1; XP_025083839.1) and HSP90 (XP_025085337.1)) was detected in AmpN (Table 2).

### 3.6. Nematode Exposure Induced Changes in Energy Metabolism

**Figure 10:**
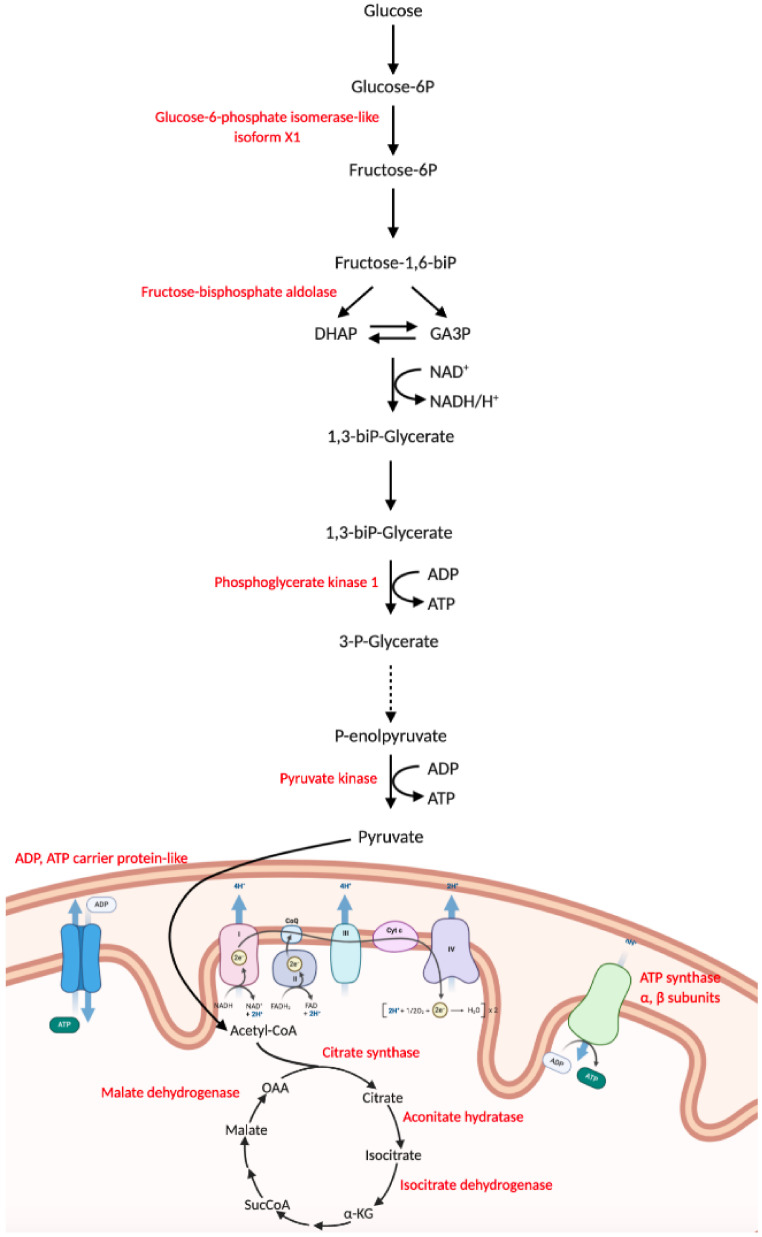
Glycolysis and respiratory pathways. The enzymes differentially expressed in AmpN samples are shown in red.

Environmental stressors can alter energy metabolism in molluscs, and on the basis of stressor type and duration, the synthesis of antioxidant enzymes and/or of HSPs can be induced or depressed with or without an energy cost [61]. Glycolysis and mitochondrial respiration represent the principal energy yielding pathways. In our experimental conditions, we observed an increased expression of several key glycolysis-related enzymes in AmpN (i.e., glucose-6-phosphate isomerase (XP_025115350.1), fructose-bisphosphate aldolase (XP_025090599.1), phosphoglycerate kinase 1 (XP_025087407.1), pyruvate kinase (XP_025081510.1), phosphoglucomutase-1 (XP_025110976.1)) (Figure 10 and Table 2). In addition, mitochondrial proteins related to ATP synthesis (i.e., ATP synthase subunits (XP_025093356.1, XP_025076941.1), ADP/ATP carrier protein (XP_025109457.1)) and enzymes involved in the citric acid cycle (i.e., citrate synthase (XP_025096557.1), aconitate hydratase (XP_025092955.1), mitochondrial isocitrate dehydrogenase [NADP]-like (XP_025089361.1) and malate dehydrogenase (XP_025089044.1)) were significantly upregulated in AmpN (Figure 10 and Table 2). These data suggested an increased energetic demand in AmpN provided by glycolytic and mitochondrial activity.

### 3.7. Nematode Exposure Influenced Cytoskeletal Dynamics 

The cytoskeleton is a highly dynamic network of filamentous proteins and plays many key roles in cell physiology. In fact, the cytoskeleton (i) is involved in the maintenance of cell shape, migration and adhesion; (ii) is responsible for organelle and protein transport; (iii) is a structural support for dividing cells; and (iv) is crucial in transducing mechanical signals throughout cells (e.g., from the ECM to the nucleus and/or among different organelles). In AmpN, we found a significant up-regulation of cytoskeletal and actin-related proteins (i.e., heavy chain myosin striated muscle-like (XP_025110638.1), alpha-actinin, sarcomeric-like isoform X1 (XP_025103379.1), paramyosin-like isoform X1 (XP_025086366.1), profilin (XP_025083894.1), gelsolin-like protein 2 (XP_025106310.1), plastin-1-like (XP_025085932.1), flotillin-2-like (XP_025092427.1)) (Table 2). Profilin influences the rate of actin polymerisation and depolymerisation [62]; gelsolin-like protein 2 is involved in actin filament assembly and disassembly processes [63]; plastin-1-like, characterised by actin binding domains, cross-links actin filaments into bundles [64]; and flotillin-2, interacting with actin, is involved in cell adhesion and movement through the ECM [65,66].

## 4. Discussion

The ampulla of *P. canaliculata* and its positioning in the circulatory system were described [37], and to date, only a few studies have thoroughly investigated the structure and function of this organ [21,38].

Here, we provided new details about the ampulla ultrastructure (see also [38]), evidencing the complex organisation of the loose connective tissue of its walls, which hosted cells of different morphologies. The ECM, a multi-molecular three-dimensional network composed of collagenous and non-collagenous proteins, is a fundamental component of multicellular organisms, with both a passive and active role in homeostasis. In particular, the ECM is a necessary scaffold for maintaining tissue structure and for cell adhesion. At the same time, the ECM provides biochemical signals to the cells, orchestrating their migration, proliferation and differentiation [67]. Along and within this complex network of fibres, different cells were observed in *P. canaliculata* ampullae. The presence of microvilli on the surface of the peripheral epithelial cells suggests a possible activity of exchange with the pericardial fluid filling the pericardial cavity in which the ampulla is contained. Embedded in the loose connective walls of the ampulla, we confirmed the presence of rhogocytes [38]. Rhogocytes have been described in different gastropod molluscs [48,68,69,70], including *P. canaliculata* [71], and have been proposed to be involved in several functions, including metabolism and/or detoxification of metal ions [50,72,73,74,75], cellular defence [68,76,77] and the production of haemocyanin [48,78,79,80,81,82], which, in its specific multimeric form, was detected in association with rhogocytes by TEM analysis in *Lymnaea stagnalis* and *Haliotis tubercolata* [49,78]. TEM observations have also documented the structure of isolated or tissue haemocyanin in *P. canaliculata* [19,83,84]. In our experimental conditions, we identified haemocyanin by proteomic analysis but not by TEM. Interestingly, in extracellular lacunae of rhogocytes, we occasionally observed material that could be haemocyanin in monomeric or oligomeric form as it has recently been proposed [71]. Further studies are necessary for understanding the synthesis, polymerisation and storage of haemocyanin in the ampulla.

At present, we do not have sufficient information to hypothesise a possible function for ampullar rhogocytes, but the presence of these cells in association with storage cells containing nitrogen-based compounds [38,51,71] further indicates that the ampulla may play a role in *P. canaliculata* physiology, maybe influencing haemolymph composition together with other organs containing nitrogen-based compounds [48,49]. The amount of uric acid has been quantified in the ampulla and other organs (e.g., lung and kidney), demonstrating a positive correlation between the amount of uric acid and the presence of urate cells [38]. These cells presented a different cytoplasmic organisation based on cyclic formation and the resorption of urate crystalloids. In the present study, the spherule-containing cells, observed within the ampulla walls of control snails, present similar traits to Stage III urate cells. For the first time, these spheres were analysed by X-ray microanalysis and selected area electron diffraction patterns. The results demonstrated that the sphere content (i.e., nitrogen, carbon, oxygen) was not organised as a crystal structure, confirming their crystalloid nature [38]. These findings support the indication that cells with spheres likely play a role in nitrogen metabolism [38,85,86,87]. In line with other reports [16,37,38,87], the present ultrastructural data suggested that ampulla activity can be influenced by, and/or contribute to, changes in haemolymph and pericardial fluid composition.

In these regards, the organ-specific proteome analysis of ampullae in control conditions and after a nematode-based immune challenge was performed.

In control conditions, the ampulla presented the proteome referable to a connective-rich and contractile organ with an extracellular environment and ECM components compatible with a space for motile cells, such as haemocytes [16] and mobile rhogocytes [48,49]. Numerous collagen-related proteins were identified. Proteins classified as collagen type I are characterised by two or more collagen triple helix repeats, necessary for correct folding and polypeptide trimerization, and one fibrillar collagen C-terminal domain, a globular domain involved in binding different substrates [88,89]. Network-forming type IV collagen consists of at least 17 collagen triple helix repeats and two C-terminal tandem repeated domains. Microfibrillar type VI collagen has three VWA domains, but the automated domain detection procedure failed to identify the collagen triple helix domain characterising this collagen in our samples [90]. In these respects, supporting the ultrastructural observations, collagen VI with VWA domains is representative of ECM components involved in cell migration and cell adhesion [67]. Similarly, the two TGFBI-related proteins identified in control ampullae could be involved in cell adhesion, migration and proliferation [53].

Proteins containing immune-related domains were also identified, reinforcing the hypothesis that ampulla functions could also be related to immune defence [16]. Proteins presenting a lectin-C type domain, serine protease inhibitor (Serpin)-domain containing proteins and a 15-hydroxyprostaglandin dehydrogenase-like protein were observed in the ampulla of unstimulated snails. In molluscs, C-type lectins are frequently described as immune components involved in non-self recognition and opsonisation [91,92,93]. Serpin-domain-containing proteins have been related to immune regulation in the Manila clam *Ruditapes philippinarum* [94] and metal binding after immune challenge in *B. glabrata* [95]. In humans, 15-hydroxyprostaglandin dehydrogenase is implied in regulating the levels of prostaglandin E 2 levels, a key mediator in different typologies of immune reactions [96], while in molluscs, a possible correlation between arachidonic acid metabolites and immunity has been suggested, but not demonstrated so far [55,97,98].

The proteome analysis of control ampullae also evidenced proteins involved in protection against oxidative/nitrosative stress. The identified enzyme list included Cu–Zn SOD and catalase- and peroxidase-like molecules, which may contribute to the maintenance of redox equilibrium. This is in line with observations that correlate the ampulla with the oxidative stress response during environmentally driven challenges [39,99]. The presence of immune-related proteins as well as of enzymes committed to the maintenance of redox equilibrium would suggest that the ampulla is potentially involved in facing oxidative stress in different occasions, e.g., during aestivation/hibernation arousal [38,39] or as a consequence of an immune challenge.

This hypothesis was further assessed by comparing the proteome of control ampullae with that of ampullae dissected from snails exposed to a nematode-based molluscicide [11]. Previous observations demonstrated that *P. canaliculata* raised a complex response towards the *P. hermaphrodita*-based molluscicide, and the immune response was influenced by both the temperature and the dose applied. The mechanisms underpinning the lethal effects of *P. hermahrodita* are still ill-defined, and they may vary on the basis of the target organism. In the land slug *Deroceras reticulatum*, the juvenile larvae of the nematode were reported to invade the dorsal integumental pouch, where they developed into self-fertilising hermaphrodites and caused the slugs’ death [12]. Another hypothesis suggested that the lethal effects could derive from the Gram-negative bacteria carried by the nematodes [13], but further evidence excludes this [14]. In *P. canaliculata*, roundworms were rarely visible in the anterior kidney and close to the gills, while they were not retrieved in the mantle or central nervous system (CNS). Consistently, none of the identified peptides in the ampulla could be related to *P. hermaphodita*.

The proteome of the ampulla of treated snails significantly changed after a 24 h exposure to the bio-pesticide. The changes in the ampulla proteome involved its connective-rich and contractile texture, as well as a vast panel of oxidative stress-related enzymes. Some ECM protein samples were differently expressed in treated snails, indicating a dynamic remodelling of the connective tissue. The remodelling of the ECM could induce the release and/or the activation of growth factors stored in the ECM [100], affecting cell proliferation and differentiation. In these respects, the levels of TGFBI were significantly reduced in treated snails. After treatment, a significant up-regulation of cytoskeletal and actin-related proteins was also observed. The variation in cytoskeletal and actin-related proteins is a common response to environmental stress and is linked to changes in cell motility and migration [63,101,102]. Recent investigations suggested that cytoskeleton modification/remodelling could affect cellular bioenergetics [103]. Consistently, ampullae from treated snails also presented a significant change in the levels of enzymes related to energy metabolism. Glycolysis and mitochondrial respiration represent the principal energy yielding pathways, and after Nemaslug^®^ exposure, several enzymes associated with ATP synthesis were more abundant. While supporting the energy demand of the cells, the increase in enzymes associated with cellular respiration could also represent a cause of oxidative stress. Moreover, it is known that environmental stressors (i.e., infection, temperature, toxic metals) can alter not only energy metabolism but also the synthesis of antioxidant enzymes and/or of HSPs, which can be either induced or suppressed on the basis of the stressing agents and their duration [61,104,105,106,107]. Imbalance between ROS production and the antioxidant defence system leads to oxidative distress [105], impairing cellular function and exerting detrimental effects on tissue components. To avoid oxidative distress, cells increase the synthesis of enzymatic and non-enzymatic antioxidant components. Nematode-treated snails presented a significant increase in both antioxidant enzymes and HSPs, which play a fundamental role in protein folding, repair, as well as in the elimination of damaged proteins. Similar results on HSP expression were obtained in the snail *Bithynia siamensis goniomphalos* when infected with the flatworm *Opisthorchis viverrine* [108].

The present findings further confirmed that *P. canaliculata* possesses a strong capacity to counteract oxidative stress, and this ability has also been related to nitrogen compound deposits observed in several organs [38,40,51,71]. The identification of molecules connected to *P. canaliculata* adaptability may prove helpful in developing single or a combination of bio-pesticides that could overcome the snail’s resilience and stop its spread in non-original countries.

## 5. Conclusions

For the first time, we correlate an ultrastructural and a proteomic analysis of *P. canaliculata* ampulla. Data indicate that this organ is characterised by loose connective tissue hosting cells of different morphologies such as rhogocytes and cells with cytoplasmic spheres containing nitrogen-based compounds. The proteome analysis evidenced proteins related to the ECM, cytoskeleton, immune system or involved in the maintenance of redox equilibrium. *P. canaliculata* reacted to the nematode-based bio-pesticide exposure with changes in expression levels of several ampullar proteins. The response to the parasitic nematode included: (i) changes in proteins associated with the cytoskeleton and ECM, suggesting a dynamic remodelling of the connective tissue; (ii) the upregulation of the antioxidant defence; (iii) the induction of stress response; and (iv) changes in the levels of proteins related to ATP-generating pathways. The collected evidence suggested that the ampulla was involved in the metabolic and stress response developed under environmental challenges, thus playing a role in the adaptive capacity of this invasive snail. On these bases, metabolic and stress-related pathways could represent a target for the efficacious and sustainable control of *P. canaliculata* spread.

## Figures and Tables

**Table 1:** List of proteins related to contractile phenotype.

Accession	Description	Accession	Description
XP_025103230.1	calponin-1-like	XP_025082010.1	myosin-2 essential light chain-like
XP_025110638.1	LQP: myosin heavy chain, striated muscle-like	XP_025087122.1	myotrophin-like
XP_025104157.1	LQP: titin-like	XP_025086366.1	paramyosin-like isoform X1
XP_025084779.1	LQP: twitchin-like	XP_025104008.1	titin homolog isoform X3
XP_025087541.1	myomodulin neuropeptides 1-like	XP_025104057.1	titin homolog isoform X1
XP_025098567.1	myophilin-like	XP_025104087.1	titin-like isoform X1
XP_025096983.1	myophilin-like	XP_025098601.1	troponin C-like isoform X1
XP_025079300.1	myosin essential light chain, striated adductor muscle-like	XP_025085471.1	troponin I-like isoform X1
XP_025095035.1	myosin regulatory light chain LC-2, mantle muscle-like isoform X2	XP_025086357.1	troponin T, skeletal muscle-like isoform X3
XP_025095034.1	myosin regulatory light chain LC-2, mantle muscle-like isoform X1		

LQP = low-quality protein.

**Table 2:** Common proteins between ampullae exposed or not exposed to the nematode with a significantly different expression level.

Accession	Description	*p*-Value	log_2_ Fold Change
XP_025110638.1	LQP: myosin heavy chain, striated muscle-like	5.89 × 10^−6^	5.289117
XP_025103379.1	alpha-actinin, sarcomeric-like isoform X1	0.000361	4.139306
XP_025090809.1	LQP: elongation factor 1-alpha-like	0.000708	3.831499
XP_025086366.1	paramyosin-like isoform X1	6.29 × 10^−7^	3.498598
XP_025104956.1	LQP: spectrin beta chain-like	0.000468	3.035385
XP_025083839.1	heat shock cognate 71 kDa protein	3.9 × 10^−5^	2.888477
XP_025098387.1	catalase-like isoform X1	0.010952	2.622116
XP_025104758.1	malate dehydrogenase, cytoplasmic-like	3.81 × 10^−5^	2.592439
XP_025106720.1	LQP: transketolase-like	0.000397	2.571769
XP_025085843.1	dihydropyrimidinase-like isoform X1	0.000429	2.521828
XP_025107274.1	LQP: glutathione S-transferase Mu 2-like	0.000396	2.341715
XP_025085667.1	lysosomal aspartic protease-like	0.024059	2.320860
XP_025078538.1	voltage-dependent anion-selective channel protein 2-like	0.012484	2.206812
XP_025082706.1	protein/nucleic acid deglycase DJ-1-like	0.002122	2.024145
XP_025092675.1	60S acidic ribosomal protein P2-like	0.006832	2.005876
XP_025113405.1	glutathione S-transferase S1-like	8.63 × 10^−5^	1.972678
XP_025082558.1	LQP: protein singed-like	0.022403	1.927912
XP_025110989.1	xylose isomerase-like	0.01412	1.833387
XP_025089044.1	malate dehydrogenase, mitochondrial-like isoform X1	0.000113	1.817043
XP_025087809.1	vinculin-like isoform X1	0.000419	1.800653
XP_025108883.1	peptidyl-prolyl cis-trans isomerase-like	1.06 × 10^−6^	1.797534
XP_025090294.1	14-3-3 protein beta/alpha-A-like	0.005248	1.763492
XP_025106211.1	glutamate receptor 1-like	8.88 × 10^−6^	1.742724
XP_025112868.1	uncharacterized protein LOC112575321 isoform X1	0.020812	1.695959
XP_025093398.1	actin-interacting protein 1-like	0.003751	1.623441
XP_025113955.1	glutathione S-transferase 1-like	0.000842	1.553176
XP_025089524.1	collagen alpha-6(VI) chain-like	0.000468	1.516933
XP_025099134.1	LQP: arginine kinase-like	0.000265	1.510341
XP_025093079.1	14-3-3 protein epsilon-like isoform X1	0.037068	1.464123
XP_025106444.1	filamin-A-like isoform X1	0.000299	1.419625
XP_025095112.1	elongation factor 1-beta-like	0.027983	1.365221
XP_025094704.1	protein disulphide-isomerase-like isoform X1	0.004507	1.359329
XP_025078628.1	LQP: neurofilament medium polypeptide-like	0.025379	1.346143
XP_025090849.1	vegetative incompatibility protein HET-E-1-like	0.001314	1.324563
XP_025099800.1	radixin-like	0.000492	1.316749
XP_025099490.1	heat shock protein 70 B2-like	0.006393	1.295692
XP_025110201.1	LQP: uncharacterized protein LOC112573811	5.36 × 10^−6^	1.269061
XP_025093885.1	LQP: uncharacterized protein LOC112563776	4.27 × 10^−5^	1.148421
XP_025090599.1	fructose-bisphosphate aldolase-like isoform X1	0.022681	1.050548
XP_025106551.1	collagen alpha-3(VI) chain-like isoform X18	0.00317	1.046923
XP_025094101.1	FK506-binding protein 2-like	0.002086	−1.02828
XP_025078843.1	glycogenin-1-like isoform X1	0.00642	−1.03570
XP_025100514.1	kinesin-like protein K39	0.003484	−1.03774
XP_025104144.1	mammalian ependymin-related protein 1-like	0.000133	−1.04629
XP_025087031.1	thymosin beta-like isoform X2	0.004896	−1.05945
XP_025080405.1	calumenin-like isoform X2	0.001715	−1.09339
XP_025109789.1	transforming growth factor-beta-induced protein ig-h3-like	0.033214	−1.18800
XP_025083419.1	small cardioactive peptides-like isoform X1	0.002106	−1.29382
XP_025096878.1	uncharacterized protein LOC112565575 isoform X1	0.002002	−1.32377
XP_025111371.1	LQP: 40S ribosomal protein S12-like	6.42 × 10^−5^	−1.38759
XP_025098601.1	troponin C-like isoform X1	0.002188	−1.41944
XP_025081260.1	collagen alpha-1(I) chain-like	0.003941	−1.51975
XP_025082853.1	uncharacterized protein LOC112557300	0.020629	−1.55671
XP_025083634.1	thioredoxin-1-like	0.034374	−1.58664
XP_025114384.1	PDZ and LIM domain protein 3-like isoform X1	0.000647	−1.60308
XP_025104157.1	LQP: titin-like	0.000772	−1.64498
XP_025085606.1	uncharacterized protein LOC112559006	9.59 × 10^−5^	−1.69730
XP_025104799.1	uncharacterized protein LOC112570529	0.001003	−1.72428
XP_025090775.1	LQP: tensin-1-like	5.75 × 10^−6^	−1.77371
XP_025087544.1	small heat shock protein p36-like	3.5 × 10^−6^	−2.04098
XP_025104087.1	titin-like isoform X1	0.000689	−2.06979
XP_025082862.1	PDZ and LIM domain protein 5-like	0.000199	−2.36176
XP_025082010.1	myosin-2 essential light chain-like	0.035777	−3.49412

Significant difference was calculated as a combination of *p*-value < 0.05 and log_2_ fold change ≥+1 or ≤−1. LQP = low-quality protein.

## Data Availability

All data generated or analysed during this study are included in this article. The mass spectrometry proteomics data presented in this study are openly available in the ProteomeXchange Consortium via the PRIDE partner repository with the dataset identifier PXD026801.

## Supplementary Materials

The following are available online at https://www.mdpi.com/article/10.3390/biology10101049/s1, Figure S1: Venn diagram of total proteins identified in ampulla exposed to nematode, Table S1: List of proteins identified in ampulla exposed (or not) to nematode, Table S2: List of proteins exclusively identified in either AmpC or AmpN samples, Table S3: List of common proteins identified in AmpC and AmpN. A label-free quantitative proteomic analysis was applied. All authors have read and agreed to the published version of the manuscript.
