# Supplementary material for: Pomacea canaliculata Ampullar Proteome: A Nematode-Based Bio-Pesticide Induces Changes in Metabolic and Stress-Related Pathways
Source: Biology (Basel). 2021 Oct 15;10(10):1049. doi: 10.3390/biology10101049 (PMC8533556; doi:10.3390/biology10101049)
Supplement: Supplementary file 1 [file biology-10-01049-s001.zip › Fig. S1.pdf]

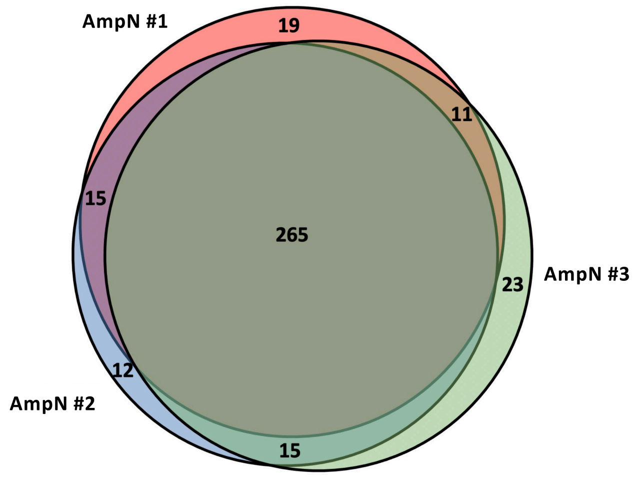

Figure S1. Venn diagram of total proteins identified in each of three biological replicates (#1, #2 and #3) of ampulla exposed to nematode (AmpN). The proteins identified in at least two biological replicates (306) were used for further analysis.
