# Supplementary material for: Pomacea canaliculata Ampullar Proteome: A Nematode-Based Bio-Pesticide Induces Changes in Metabolic and Stress-Related Pathways
Source: Biology (Basel). 2021 Oct 15;10(10):1049. doi: 10.3390/biology10101049 (PMC8533556; doi:10.3390/biology10101049)
Supplement: Supplementary file 1 [file biology-10-01049-s001.zip › Table S1.pdf]

Table S1

			SCORE						Num. of significant unique sequences						emPAI					
Accession	Description	MW (kDA)	AmpA	AmpB	AmpC	AmpNA	AmpNB	AmpNC	AmpA	AmpB	AmpC	AmpNA	AmpNB	AmpNC	AmpA	AmpB	AmpC	AmpNA	AmpNB	AmpNC
XP_025089564.1	LQP: hemocyanin G-type, units Oda to Odg-like	393,218	11247	11379	10939	21281	21961	21343	97	96	91	108	109	111	1,59	1,59	1,41	2,16	2,13	2,13
XP_025089796.1	hemocyanin G-type, units Oda to Odg-like	392,973	10784	11259	11014	17786	18842	18784	104	100	93	112	111	110	1,77	1,68	1,44	2,16	2,13	2,19
XP_025106444.1	filamin-A-like isoform X1	280,77	4352	3951	3992	8507	8514	8628	62	57	57	103	96	102	1,14	0,99	1,02	2,66	2,31	2,65
XP_025112424.1	actin, adductor muscle	42,107	3918	3650	3964	5767	5761	5986	7	7	6	8	8	7	4,33	4,78	3,93	6,97	8,32	5,78
XP_025110090.1	actin, cytoplasmic	42,124	2076	2043	2204	3508	3425	3667	3	3	3	4	4	4	2,05	2,58	2,31	2,89	3,2	2,88
XP_025110638.1	LQP: myosin heavy chain, striated muscle-like	226,077	148	135	120	4911	5194	4741	2	3	4	64	65	67	0,03	0,05	0,06	1,62	1,65	1,73
XP_025089549.1	cartilage matrix protein-like	48,649	4041	4422	4003	4392	4435	3909	15	16	16	14	14	16	2,72	3,57	2,72	2,73	2,98	3,58
XP_025102035.1	15-hydroxyprostaglandin dehydrogenase [NAD(+)]-like	30,797	2772	2580	2539	4148	4118	4300	17	17	17	16	16	17	6,05	6,05	6,05	6,88	6,86	7,76
XP_025077978.1	filamin-A-like isoform X3	90,568	1261	1191	1231	0	0	0	27	26	26	0	0	0	1,84	1,74	1,64	0	0	0
XP_025078481.1	murinoglobulin-1-like isoform X8	184,938	3161	3190	2764	3416	3609	3385	46	45	42	47	51	44	1,41	1,28	1,2	1,51	1,69	1,28
XP_025086366.1	paramyosin-like isoform X1	102,022	660	579	458	3054	3073	3079	6	7	4	34	34	34	0,22	0,26	0,14	2,09	2,09	2,09
XP_025115938.1	tubulin beta-4B chain-like	50,189	0	0	0	2366	2357	2537	0	0	0	2	2	2	0	0	0	3,09	2,82	3,08
XP_025109819.1	tubulin alpha-2/alpha-4 chain	50,856	0	0	0	2408	2336	2276	0	0	0	2	2	2	0	0	0	2,08	2,29	2,08
XP_025086180.1	tubulin alpha-1A chain-like	50,746	0	0	0	2077	2029	1981	0	0	0	3	3	3	0	0	0	1,71	1,89	1,89
XP_025093240.1	tubulin alpha chain, testis-specific-like	50,914	0	0	0	1821	1772	1681	0	0	0	4	4	3	0	0	0	1,88	2,07	1,69
XP_025107285.1	tubulin alpha-8 chain-like	51,485	0	0	0	1732	1706	1691	0	0	0	5	4	5	0	0	0	1,67	1,66	1,66
XP_025093885.1	LQP: uncharacterized protein LOC112563776	149,309	727	910	863	2151	2101	2106	19	20	19	34	32	32	0,54	0,57	0,54	1,17	1,07	1,07
XP_025103230.1	calponin-1-like	45,774	3404	3092	3152	2008	1924	1894	25	24	25	22	22	22	8,05	7,41	8,05	5,29	4,41	4,83
XP_025083091.1	enolase-like	47,282	761	810	740	1997	2154	2187	17	18	16	20	20	20	2,59	2,86	2,35	3,46	3,45	3,45
XP_025106551.1	collagen alpha-3(VI) chain-like isoform X18	286,004	941	777	571	1983	2064	2019	16	16	12	28	26	22	0,21	0,21	0,15	0,4	0,36	0,3
XP_025106211.1	glutamate receptor 1-like	56,36	346	291	298	1719	1909	1957	7	8	8	17	17	17	0,52	0,61	0,61	1,76	1,76	1,76
XP_025082312.1	glyceraldehyde-3-phosphate dehydrogenase-like isoform X1	39,33	1497	1468	1329	1714	1677	1676	12	12	13	15	14	15	1,78	1,78	2,03	2,6	2,3	2,59
XP_025104758.1	malate dehydrogenase, cytoplasmic-like	36,887	539	593	473	1677	1563	1494	5	7	7	19	18	18	0,57	0,89	0,89	4,63	4,13	4,13
XP_025087209.1	transgelin-2-like	18,649	1080	1133	937	1586	1749	1995	10	11	9	11	11	12	4,85	5,98	3,9	7,35	8,94	7,33
XP_025114701.1	peptidyl-prolyl cis-trans isomerase B-like	23,806	1115	1079	978	1565	1663	1483	9	10	8	5	6	6	3,65	4,34	2,51	5,16	6,06	4,34
XP_025114702.1	LQP: peptidyl-prolyl cis-trans isomerase B-like	23,999	0	0	0	1519	1618	1582	0	0	0	5	5	5	0	0	0	3	3,58	3,58
XP_025087809.1	vinculin-like isoform X1	121,601	409	385	426	1538	1360	1428	8	5	7	18	17	19	0,25	0,15	0,22	0,65	0,61	0,7
XP_025099134.1	LQP: arginine kinase-like	81,474	341	306	330	1509	1518	1562	5	4	4	8	8	8	0,39	0,28	0,28	0,79	0,79	0,86
XP_025099135.1	LQP: arginine kinase-like	140,972	204	0	0	995	900	999	2	0	0	4	4	4	0,13	0	0	0,27	0,27	0,3
XP_025083839.1	heat shock cognate 71 kDa protein	71,517	154	239	157	1479	1541	1444	4	6	2	18	17	18	0,27	0,39	0,15	1,83	1,69	1,82
XP_025112799.1	endoplasmic reticulum chaperone BiP	74,114	0	0	0	1135	1244	1100	0	0	0	12	14	14	0	0	0	0,98	1,17	1,17
XP_025099490.1	heat shock protein 70 B2-like	70,414	101	128	146	566	685	531	2	2	2	4	6	5	0,15	0,15	0,15	0,27	0,4	0,33
XP_025095035.1	myosin regulatory light chain LC-2, mantle muscle-like isoform X2	19,236	3441	3342	3621	0	0	0	2	2	2	0	0	0	50,86	50,87	29,99	0	0	0
XP_025103379.1	alpha-actinin, sarcomeric-like isoform X1	104,054	133	77	78	1327	1430	1476	2	2	3	27	27	22	0,07	0,07	0,1	1,41	1,41	1,05
XP_025113955.1	glutathione S-transferase 1-like	22,631	392	359	351	1317	1238	1397	4	5	4	6	6	7	1,78	2,22	1,78	4,8	4,79	5,7
XP_025113405.1	glutathione S-transferase S1-like	48,76	362	399	430	1038	941	1024	3	4	3	8	7	8	0,41	0,51	0,41	1,63	1,45	1,63
XP_025098567.1	myophilin-like	19,402	1850	1662	1657	1310	1337	1416	11	10	10	12	10	11	11,76	6,67	9,77	8,11	5,47	6,67
XP_025081459.1	talin-1-like isoform X1	282,925	0	0	0	1296	1155	1281	0	0	0	32	32	36	0	0	0	0,47	0,47	0,54
XP_025113391.1	LQP: spectrin alpha chain-like	283,325	0	0	116	1261	1246	1377	0	0	2	34	38	38	0	0	0,02	0,5	0,58	0,58
XP_025095804.1	uncharacterized protein LOC112564910	25,874	1076	1186	1150	1242	1300	1222	8	8	10	9	9	9	2,18	2,18	3,11	2,62	2,62	2,62
XP_025114751.1	synaptic vesicle membrane protein VAT-1 homolog-like	45,088	654	641	536	1234	1187	1079	15	14	12	16	18	18	2,06	1,84	1,44	2,3	2,82	2,82
XP_025104956.1	LQP: spectrin beta chain-like	282,322	258	225	177	1233	1186	868	5	6	4	31	34	26	0,06	0,07	0,05	0,45	0,51	0,37
XP_025093356.1	ATP synthase subunit beta, mitochondrial-like	63,466	0	0	0	1117	923	936	0	0	0	17	15	14	0	0	0	1,47	1,22	1,1
XP_025090809.1	LQP: elongation factor 1-alpha-like	50,341	102	79	0	1112	1086	1114	2	3	0	14	13	14	0,14	0,22	0	1,55	1,38	1,54
XP_025094704.1	protein disulfide-isomerase-like isoform X1	56,331	372	406	361	1108	1358	1052	11	11	10	17	20	18	0,93	0,93	0,82	1,77	2,51	1,93
XP_025093079.1	14-3-3 protein epsilon-like isoform X1	38,999	0	455	445	1032	1068	1054	0	8	7	10	11	10	0	1,17	1,17	1,81	2,06	2,06
XP_025090294.1	14-3-3 protein beta/alpha-A-like	29,423	99	82	138	315	312	356	2	2	3	7	6	7	0,57	0,4	0,76	1,78	1,48	2,11
XP_025091015.1	collagen alpha-5(VI) chain-like isoform X2	49,616	0	522	0	969	1110	1068	0	9	0	13	13	13	0	0,84	0	1,42	1,41	1,41
XP_025076319.1	phosphoenolpyruvate carboxykinase [GTP]-like	74,042	0	0	0	923	885	867	0	0	0	16	16	16	0	0	0	1,08	1,07	1,07
XP_025080405.1	calumenin-like isoform X2	37,646	2175	2471	2776	901	1003	1017	5	4	5	3	2	3	6,74	5,48	6,08	2,81	2,48	2,48

XP_025080404.1	calumenin-B-like isoform X1	38,56	0	0	2638	0	991	975	0	0	2	0	2	2	0	0	3,78	0	2,38	2,1
XP_025078041.1	LQP: collagen alpha-5(VI) chain-like	45,287	574	574	462	892	702	734	14	10	9	13	11	11	1,82	1,1	0,95	1,63	1,26	1,26
XP_025106720.1	LQP: transketolase-like	68,647	239	122	118	828	733	509	6	4	2	16	14	14	0,34	0,22	0,1	1,31	1,09	1,09
XP_025109080.1	calreticulin-like	47,418	449	572	408	797	629	796	12	11	10	11	11	11	1,34	1,18	1,03	1,18	1,18	1,18
XP_025078101.1	translation elongation factor 2-like	95,71	0	0	0	766	788	783	0	0	0	20	19	21	0	0	0	1,03	0,96	1,1
XP_025099080.1	alpha-crystallin A chain-like	19,893	1140	1265	1303	749	740	803	7	7	7	6	6	6	2,78	2,78	2,78	2,2	2,2	2,2
XP_025096925.1	alpha-crystallin B chain-like	19,016	246	339	234	0	0	92	3	4	4	0	0	2	1	1,38	1,38	0	0	0,68
XP_025096926.1	protein lethal(2)essential for life-like	17,506	179	165	196	50	67	74	3	3	3	2	2	2	1,12	1,12	1,12	0,76	0,75	0,75
XP_025077321.1	LQP: thrombospondin type-1 domain-containing protein 4-like	89,636	1437	1076	1381	747	661	617	15	13	13	12	14	12	0,76	0,63	0,63	0,57	0,69	0,57
XP_025111140.1	far upstream element-binding protein 3-like	19,317	1361	1153	1381	747	925	771	10	9	8	7	8	7	5,54	5,54	4,51	3,66	4,51	2,92
XP_025110201.1	LQP: uncharacterized protein LOC112573811	68,834	137	120	77	728	703	491	4	4	4	8	8	8	0,22	0,22	0,22	0,48	0,48	0,48
XP_025080024.1	serine protease inhibitor 2.1-like isoform X1	45,028	540	579	452	726	635	787	3	5	3	4	5	4	0,56	0,81	0,68	1,11	1,27	0,96
XP_025080031.1	serine protease inhibitor 2.1-like	37,532	499	429	417	548	534	656	2	2	2	2	2	2	0,87	0,71	0,87	1,44	1,44	1,44
XP_025080019.1	leukocyte elastase inhibitor-like	50,365	0	0	0	407	441	338	0	0	0	3	3	2	0	0	0	0,4	0,4	0,4
XP_025104019.1	coiled-coil domain-containing protein 141-like isoform X1	170,252	0	0	0	700	606	720	0	0	0	19	19	17	0	0	0	0,46	0,46	0,4
XP_025079300.1	myosin essential light chain, striated adductor muscle-like	17,705	667	690	748	694	707	576	10	10	10	9	9	9	5,37	5,37	5,37	4,3	5,37	5,37
XP_025082558.1	LQP: protein singed-like	56,229	178	264	243	664	583	341	6	5	5	15	13	10	0,43	0,35	0,35	1,77	1,31	0,82
XP_025090599.1	fructose-bisphosphate aldolase-like isoform X1	46,254	349	234	162	640	634	680	5	4	4	6	6	7	0,66	0,34	0,34	0,92	0,92	0,66
XP_025098992.1	superoxide dismutase [Cu-Zn]-like	15,772	1462	1551	1407	626	539	421	6	6	6	6	6	5	2,48	2,48	2,48	2,48	2,48	1,83
XP_025076941.1	ATP synthase subunit alpha, mitochondrial-like	62,838	0	0	0	622	625	634	0	0	0	15	13	14	0	0	0	1,24	1,01	1,12
XP_025106309.1	gelsolin-like protein 2 isoform X2	41,889	0	0	0	608	660	576	0	0	0	12	12	13	0	0	0	1,84	1,83	1,83
XP_025095794.1	LQP: 60 kDa heat shock protein, mitochondrial-like	62,81	0	126	0	601	533	511	0	2	0	10	10	10	0	0,11	0	0,71	0,71	0,71
XP_025083507.1	uncharacterized protein LOC112557719	37,077	1151	1311	1305	590	578	486	10	9	10	9	8	7	1,7	1,25	1,7	1,26	1,25	0,88
XP_025089044.1	malate dehydrogenase, mitochondrial-like isoform X1	39,428	187	153	107	586	642	523	6	5	4	12	12	11	0,67	0,53	0,41	1,78	1,77	1,55
XP_025086134.1	hemicentin-2-like isoform X1	173,937	1822	1915	1838	586	542	588	30	29	26	15	14	17	0,79	0,79	0,66	0,34	0,31	0,39
XP_025097192.1	NADP-dependent malic enzyme-like	67,178	536	548	437	572	552	495	11	10	8	11	9	10	0,74	0,65	0,49	0,74	0,57	0,65
XP_025085337.1	heat shock protein HSP 90-alpha-like isoform X1	80,052	0	0	0	568	606	656	0	0	0	13	11	13	0	0	0	0,81	0,66	0,81
XP_025107756.1	endoplasmin-like	90,638	0	0	0	443	375	463	0	0	0	8	8	9	0	0	0	0,4	0,4	0,45
XP_025084779.1	LQP: twitchin-like	523,956	604	600	602	525	516	629	19	22	14	16	16	22	0,13	0,15	0,1	0,11	0,11	0,15
XP_025107717.1	rab GDP dissociation inhibitor alpha-like	51,802	0	0	0	525	609	706	0	0	0	14	12	13	0	0	0	1,48	1,18	1,32
XP_025083164.1	neprilysin-4-like	83,182	0	0	0	524	524	524	0	0	0	14	13	16	0	0	0	0,84	0,77	1
XP_025108883.1	peptidyl-prolyl cis-trans isomerase-like	22,78	86	90	142	522	613	574	2	2	2	4	4	4	0,34	0,34	0,34	1,07	1,07	1,07
XP_025104607.1	histone H3-like	27,463	0	0	0	507	527	501	0	0	0	6	7	6	0	0	0	1,07	1,34	1,07
XP_025113499.1	PDZ and LIM domain protein 7-like isoform X1	71,683	343	442	416	497	583	587	8	8	8	12	10	11	0,46	0,46	0,46	0,76	0,6	0,68
XP_025089563.1	clathrin heavy chain 1	193,968	0	0	0	496	383	352	0	0	0	10	10	8	0	0	0	0,19	0,19	0,15
XP_025110182.1	LQP: peroxidasin-like	153,735	0	0	0	495	516	464	0	0	0	15	14	13	0	0	0	0,39	0,36	0,33
XP_025089511.1	collagen alpha-6(VI) chain-like	69,724	314	324	262	475	502	445	9	8	7	11	11	11	0,55	0,47	0,4	0,7	0,7	0,7
XP_025086357.1	troponin T, skeletal muscle-like isoform X3	39,049	303	216	242	472	460	558	8	7	8	9	9	10	0,99	0,82	0,99	1,17	1,16	1,36
XP_025098387.1	catalase-like isoform X1	59,457	120	67	0	466	386	457	4	2	0	8	9	10	0,25	0,12	0	0,57	0,66	0,76
XP_025092500.1	LQP: galectin-4-like	64,718	0	0	0	454	370	339	0	0	0	10	8	7	0	0	0	0,68	0,52	0,44
XP_025085843.1	dihydropyrimidinase-like isoform X1	72,895	354	306	352	446	488	407	2	2	2	10	8	9	0,1	0,1	0,1	0,59	0,45	0,52
XP_025109457.1	ADP,ATP carrier protein-like	34,722	0	0	0	444	394	316	0	0	0	9	7	8	0	0	0	1,38	0,96	1,16
XP_025101259.1	serine/threonine-protein phosphatase 2A 65 kDa regulatory subunit A alpha isoform-like isoform	67,282	0	0	0	435	430	360	0	0	0	7	7	7	0	0	0	0,42	0,42	0,42
XP_025084336.1	uncharacterized protein LOC112558220	10,169	1028	1113	1254	430	389	376	5	5	5	5	5	5	3,81	3,81	3,81	3,82	3,81	3,81
XP_025107274.1	LQP: glutathione S-transferase Mu 2-like	25,021	193	145	124	423	476	430	3	2	2	8	7	7	0,49	0,3	0,3	1,9	1,54	1,54
XP_025089430.1	uncharacterized protein LOC112561269	24,887	205	263	160	409	408	338	4	4	5	6	6	6	0,71	0,71	0,95	1,23	1,23	1,23
XP_025107962.1	uncharacterized protein LOC112572474	24,79	578	599	576	400	377	460	4	4	4	3	3	3	0,71	0,71	0,71	0,5	0,5	0,5
XP_025107262.1	uncharacterized protein LOC112572003	16,793	220	0	243	394	421	321	2	0	2	3	2	2	0,48	0	0,48	0,8	0,48	0,48
XP_025080714.1	thiamin pyrophosphokinase 1-like isoform X1	30,609	261	183	231	391	332	367	6	5	6	6	6	5	0,92	0,72	0,92	0,92	0,92	0,72
XP_025092669.1	cartilage matrix protein-like	46,021	798	644	711	389	421	429	13	12	12	10	9	10	1,58	1,4	1,4	1,08	0,93	1,07
XP_025087032.1	uncharacterized protein LOC112559814 isoform X3	27,047	0	1263	1334	0	0	0	0	2	2	0	0	0	0	4,58	3,94	0	0	0
XP_025087031.1	thymosin beta-like isoform X2	27,005	1063	1011	1107	317	364	348	2	2	2	2	2	2	3,97	5,36	3,97	2,04	1,68	2,03

XP_025087033.1	thymosin beta-like isoform X4	22,481	742	748	742	0	0	0	2	2	2	0	0	0	4,04	5,76	4,04	0	0	0
XP_025104976.1	histone H2B, gonadal	13,46	0	90	0	366	428	421	0	2	0	3	4	3	0	0,62	0	1,07	1,64	1,07
XP_025109714.1	LQP: glycogen debranching enzyme-like	192,004	0	0	0	361	390	369	0	0	0	10	9	9	0	0	0	0,19	0,17	0,17
XP_025115912.1	purine nucleoside phosphorylase-like	31,682	0	0	128	358	312	369	0	0	2	6	5	6	0	0	0,23	0,89	0,69	0,88
XP_025078405.1	microtubule-associated protein futsch-like isoform X1	185,943	820	854	795	355	450	0	16	16	15	9	11	0	0,36	0,34	0,34	0,18	0,22	0
XP_025078538.1	voltage-dependent anion-selective channel protein 2-like	30,26	86	52	0	355	312	293	2	2	0	5	5	4	0,25	0,25	0	0,74	0,73	0,55
XP_025100514.1	kinesin-like protein K39	80,94	648	927	797	353	338	250	11	11	11	6	5	6	0,58	0,58	0,65	0,29	0,23	0,28
XP_025082853.1	uncharacterized protein LOC112557300	11,229	1115	1063	1080	348	231	277	4	4	5	2	2	2	2,16	2,16	3,21	0,78	0,78	0,78
XP_025103811.1	glycogen phosphorylase, muscle form-like	97,71	0	0	0	343	356	285	0	0	0	9	9	7	0	0	0	0,37	0,37	0,27
XP_025077535.1	protein disulfide-isomerase A3-like	62,564	0	0	0	336	357	356	0	0	0	5	8	7	0	0	0	0,31	0,54	0,46
XP_025104157.1	LQP: titin-like	1219,355	2328	2331	2218	333	605	597	46	42	40	11	16	12	0,14	0,12	0,12	0,03	0,05	0,03
XP_025112526.1	rho GDP-dissociation inhibitor 1-like	23,343	291	207	372	331	271	316	6	5	7	6	6	7	1,35	1,04	1,71	1,35	1,35	1,71
XP_025087821.1	1,4-alpha-glucan-branching enzyme-like isoform X1	90,914	0	0	0	330	331	384	0	0	0	11	7	9	0	0	0	0,51	0,3	0,4
XP_025081304.1	glyoxylate reductase/hydroxypyruvate reductase-like isoform X1	36,76	0	0	0	329	325	355	0	0	0	5	5	5	0	0	0	0,58	0,58	0,58
XP_025087544.1	small heat shock protein p36-like	37,251	2231	2269	2148	326	344	304	22	22	21	9	11	10	6,88	6,88	6,2	1,25	1,68	1,45
XP_025099800.1	radixin-like	70,015	127	152	119	326	363	306	4	5	4	9	8	9	0,21	0,27	0,21	0,54	0,47	0,54
XP_025085606.1	uncharacterized protein LOC112559006	212,918	2622	2638	2507	318	329	264	26	25	25	9	8	8	0,54	0,49	0,51	0,15	0,14	0,14
XP_025091980.1	reticulon-1-like isoform X1	42,468	620	624	528	309	245	226	3	3	2	4	4	3	0,27	0,27	0,17	0,37	0,37	0,27
XP_025089524.1	collagen alpha-6(VI) chain-like	126,544	142	102	142	309	326	354	4	3	4	8	9	9	0,11	0,08	0,11	0,24	0,27	0,27
XP_025086690.1	myophilin-like	23,143	0	0	0	308	213	205	0	0	0	3	4	4	0	0	0	0,54	0,78	0,78
XP_025112685.1	LIM domain-containing protein WLIM2b-like	9,086	440	393	425	299	240	302	5	5	7	4	4	4	4,76	4,76	10,6	3,06	3,06	3,06
XP_025081483.1	uncharacterized protein LOC112556562	27,663	0	0	0	298	282	314	0	0	0	4	4	5	0	0	0	0,62	0,62	0,83
XP_025100353.1	calmodulin, striated muscle-like isoform X1	12,158	0	0	478	297	332	399	0	0	5	3	3	3	0	0	3,91	1,22	1,22	1,22
XP_025082249.1	glyoxylate reductase/hydroxypyruvate reductase-like	36,919	0	0	0	294	322	281	0	0	0	8	10	7	0	0	0	1,07	1,71	0,88
XP_025080083.1	retinal dehydrogenase 2-like	54,365	83	0	0	293	324	346	2	0	0	10	8	8	0,13	0	0	0,86	0,64	0,64
XP_025092049.1	actin-depolymerizing factor 2-like	17,168	211	266	234	293	256	166	5	6	5	5	4	4	1,6	2,14	1,6	1,6	1,15	1,15
XP_025106061.1	LQP: UTP--glucose-1-phosphate uridylyltransferase-like	57,313	0	0	0	292	298	264	0	0	0	7	6	7	0	0	0	0,51	0,42	0,51
XP_025081499.1	eukaryotic translation initiation factor 5A-1-like isoform X1	18,679	609	579	585	290	290	250	8	8	7	6	5	5	3,11	3,9	3,11	1,89	1,42	1,42
XP_025094027.1	uncharacterized protein LOC112563852 isoform X1	284,183	462	498	390	288	336	333	8	7	7	9	9	10	0,1	0,09	0,09	0,11	0,11	0,13
XP_025087407.1	phosphoglycerate kinase 1-like	44,72	0	0	0	286	320	354	0	0	0	10	7	12	0	0	0	1,12	0,69	1,46
XP_025105842.1	enolase-phosphatase E1-like	35,07	381	359	277	283	302	138	4	3	3	5	4	3	0,46	0,33	0,33	0,61	0,46	0,33
XP_025095713.1	guanine nucleotide-binding protein subunit beta-2-like 1	36,029	0	0	0	282	146	164	0	0	0	4	3	3	0	0	0	0,45	0,32	0,32
XP_025094983.1	uncharacterized protein ZK1073.1-like isoform X1	42,892	0	0	0	279	196	230	0	0	0	6	6	6	0	0	0	0,6	0,6	0,6
XP_025088756.1	LQP: carbonyl reductase [NADPH] 1-like	30,197	0	0	0	277	232	218	0	0	0	7	6	7	0	0	0	1,17	0,94	1,17
XP_025096983.1	myophilin-like	21,965	122	97	125	274	273	290	3	4	4	6	4	3	0,57	0,83	0,83	1,47	0,83	0,57
XP_025097225.1	40S ribosomal protein S4-like	29,934	0	0	0	266	217	196	0	0	0	6	7	5	0	0	0	0,95	1,18	0,74
XP_025092675.1	60S acidic ribosomal protein P2-like	11,869	267	306	214	263	298	257	2	2	3	5	5	6	0,72	0,72	1,26	2,91	2,9	4,12
XP_025094855.1	LQP: aspartate aminotransferase, cytoplasmic-like	47,33	40	0	0	263	255	129	2	0	0	5	9	8	5	0,15	0	0	0,9	0,77	0,43
XP_025115754.1	aspartate aminotransferase, mitochondrial-like	47,84	0	0	0	113	159	58	0	0	0	4	3	3	0	0	0	0,32	0,23	0,23
XP_025110989.1	xylose isomerase-like	51,235	52	72	31	262	193	222	2	2	2	7	6	4	0,14	0,14	0,14	0,58	0,48	0,3
XP_025088846.1	hsc70-interacting protein-like isoform X1	40,873	0	131	0	249	234	210	0	2	0	4	4	5	0	0,18	0	0,39	0,39	0,51
XP_025082709.1	SH3 domain-binding glutamic acid-rich-like protein 3	9,715	702	710	747	246	228	275	4	4	4	3	3	2	2,71	2,71	2,71	1,67	1,67	0,93
XP_025109846.1	40S ribosomal protein S7-like	22,413	0	0	0	238	132	150	0	0	0	6	4	5	0	0	0	1,43	0,81	1,09
XP_025112033.1	LQP: retrograde protein of 51 kDa-like	65,635	0	0	0	237	133	139	0	0	0	7	5	6	0	0	0	0,43	0,29	0,36
XP_025089532.1	60S acidic ribosomal protein P0-like	34,923	0	0	0	235	216	202	0	0	0	5	6	5	0	0	0	0,61	0,78	0,61
XP_025098733.1	chloride intracellular channel protein 4-like	34,533	0	0	0	159	241	176	0	0	0	4	4	4	0	0	0	0,79	0,79	0,97
XP_025087966.1	copper transport protein ATOX1-like	7,922	611	614	689	231	236	208	3	3	3	2	2	2	2,32	2,32	2,32	1,23	1,23	1,23
XP_025107348.1	superoxide dismutase [Cu-Zn]-like	25,462	81	102	63	230	192	143	2	2	2	2	2	2	0,3	0,3	0,3	0,3	0,3	0,3
XP_025090384.1	mediator of RNA polymerase II transcription subunit 15-like isoform X1	77,172	0	0	0	229	266	290	0	0	0	5	7	6	0	0	0	0,25	0,36	0,3
XP_025079145.1	eukaryotic initiation factor 4A-I-like isoform X1	53,893	0	0	0	229	281	193	0	0	0	5	6	6	0	0	0	0,37	0,45	0,45
XP_025082600.1	basement membrane-specific heparan sulfate proteoglycan core protein-like isoform X1	529,516	0	0	0	225	213	298	0	0	0	10	7	8	0	0	0	0,07	0,05	0,05
XP_025085667.1	lysosomal aspartic protease-like	44,002	103	83	0	220	144	155	2	2	0	6	5	4	0,16	0,16	0	0,58	0,46	0,36

XP_025104087.1	titin-like isoform X1	118,168	1048	1050	888	220	182	192	14	15	11	4	3	3	0,49	0,54	0,37	0,12	0,09	0,09
XP_025114842.1	uncharacterized protein LOC112576527 isoform X10	37,345	0	0	0	219	210	250	0	0	0	5	4	4	0	0	0	0,57	0,43	0,43
XP_025083191.1	peptidyl-prolyl cis-trans isomerase B-like isoform X1	26,823	0	0	0	214	171	164	0	0	0	6	5	5	0	0	0	1,1	0,86	0,86
XP_025078628.1	LQP: neurofilament medium polypeptide-like	27,776	80	101	72	214	215	168	2	2	2	4	5	3	0,27	0,27	0,27	0,62	0,82	0,43
XP_025089867.1	adenylyl cyclase-associated protein 1-like isoform X1	57,322	0	0	0	213	155	129	0	0	0	11	7	7	0	0	0	0,91	0,51	0,51
XP_025106788.1	uncharacterized protein LOC112571747	17,699	127	179	86	212	159	135	3	3	2	4	2	2	0,75	0,75	0,45	1,11	0,45	0,45
XP_025109789.1	transforming growth factor-beta-induced protein ig-h3-like	86,247	259	273	224	211	175	139	7	5	4	3	2	2	0,32	0,22	0,17	0,12	0,08	0,08
XP_025085574.1	60S ribosomal protein L9-like	21,396	0	0	0	208	159	162	0	0	0	3	3	2	0	0	0	0,59	0,59	0,36
XP_025083177.1	sorbitol dehydrogenase-like	37,996	0	0	0	207	179	111	0	0	0	6	5	5	0	0	0	0,86	0,7	0,55
XP_025097457.1	sorbitol dehydrogenase-like	37,818	0	0	0	62	104	0	0	0	0	2	2	0	0	0	0	0,3	0,3	0
XP_025103728.1	ras-related protein Rab-1A	23,017	0	0	0	207	165	117	0	0	0	3	3	2	0	0	0	1,06	1,06	0,78
XP_025112474.1	LQP: 40S ribosomal protein S5-like	23,437	0	0	0	207	204	210	0	0	0	3	3	3	0	0	0	0,53	0,53	0,53
XP_025096878.1	uncharacterized protein LOC112565575 isoform X1	270,574	949	807	889	207	190	206	11	11	11	6	3	4	0,16	0,16	0,15	0,08	0,04	0,05
XP_025080479.1	6-phosphogluconate dehydrogenase, decarboxylating-like	53,107	0	0	0	207	186	149	0	0	0	4	3	3	0	0	0	0,29	0,21	0,21
XP_025092026.1	protein disulfide-isomerase A6 homolog	48,288	0	0	0	205	194	205	0	0	0	3	3	2	0	0	0	0,23	0,23	0,15
XP_025105844.1	LQP: saccharopine dehydrogenase-like oxidoreductase	47,947	0	0	0	203	186	200	0	0	0	4	4	5	0	0	0	0,32	0,32	0,42
XP_025115350.1	glucose-6-phosphate isomerase-like isoform X1	67,687	0	0	0	197	174	128	0	0	0	5	5	4	0	0	0	0,28	0,28	0,22
XP_025085471.1	troponin I-like isoform X1	45,356	0	0	0	194	184	202	0	0	0	6	5	5	0	0	0	0,56	0,45	0,45
XP_025078437.1	40S ribosomal protein S6	28,562	0	0	0	191	181	180	0	0	0	4	3	4	0	0	0	0,6	0,42	0,6
XP_025098343.1	T-complex protein 1 subunit beta-like isoform X1	58,366	0	0	0	191	169	154	0	0	0	2	2	2	0	0	0	0,12	0,12	0,12
XP_025082706.1	protein/nucleic acid deglycase DJ-1-like	19,525	78	67	54	191	181	153	2	2	2	5	5	6	0,4	0,4	0,4	1,33	1,33	1,75
XP_025089361.1	isocitrate dehydrogenase [NADP], mitochondrial-like	51,216	0	0	0	189	187	177	0	0	0	4	5	5	0	0	0	0,3	0,39	0,39
XP_025088246.1	40S ribosomal protein S13	17,21	0	0	0	188	198	173	0	0	0	4	4	4	0	0	0	1,15	1,15	1,15
XP_025089277.1	cofilin-like	17,015	139	159	148	188	168	248	2	2	2	3	2	3	0,47	0,47	0,47	0,79	0,47	0,79
XP_025098601.1	troponin C-like isoform X1	17,508	741	800	648	187	204	202	8	7	7	3	4	4	3,48	2,71	2,71	0,76	1,12	1,12
XP_025095375.1	uncharacterized protein LOC112564634	43,121	0	0	0	186	152	143	0	0	0	4	4	4	0	0	0	0,37	0,37	0,37
XP_025106684.1	uncharacterized protein LOC112571691	18,724	203	150	155	185	192	149	5	5	4	4	4	3	1,41	1,41	1,02	1,02	1,02	0,69
XP_025090849.1	vegetative incompatibility protein HET-E-1-like	45,553	121	75	72	183	146	131	4	3	2	6	6	6	0,34	0,25	0,16	0,56	0,56	0,56
XP_025112159.1	cAMP-dependent protein kinase regulatory subunit isoform X1	47,823	0	0	0	180	169	230	0	0	0	5	4	4	0	0	0	0,42	0,32	0,32
XP_025104082.1	barrier-to-autointegration factor-like	10,537	292	294	241	180	165	156	2	2	2	2	2	2	0,84	0,84	0,84	0,84	0,84	0,84
XP_025109348.1	transitional endoplasmic reticulum ATPase	89,731	0	0	0	178	225	148	0	0	0	3	6	3	0	0	0	0,12	0,25	0,12
XP_025093398.1	actin-interacting protein 1-like	66,487	141	140	93	171	187	147	4	4	2	7	8	9	0,22	0,22	0,11	0,43	0,5	0,58
XP_025096543.1	ADP-ribosylation factor 2 isoform X1	21,162	0	0	0	170	151	179	0	0	0	4	3	4	0	0	0	0,87	0,6	0,87
XP_025095112.1	elongation factor 1-beta-like	23,503	149	152	105	170	135	179	2	2	2	5	3	4	0,33	0,33	0,33	1,02	0,53	0,76
XP_025082954.1	uncharacterized protein LOC112557363	9,312	413	404	388	169	193	0	4	3	3	3	3	0	4,54	1,79	1,79	1,79	1,79	0
XP_025115033.1	adenosylhomocysteinase-like	48,265	0	0	0	168	267	210	0	0	0	6	8	6	0	0	0	0,52	0,74	0,52
XP_025106186.1	40S ribosomal protein SA-like	33,683	0	0	0	167	185	262	0	0	0	3	4	4	0	0	0	0,35	0,49	0,49
XP_025088066.1	ubiquitin carboxyl-terminal hydrolase-like	25,395	0	0	0	166	100	0	0	0	0	2	2	0	0	0	0	0,3	0,3	0
XP_025077458.1	LQP: fibrillin-2-like	333,923	0	99	77	166	1150	144	0	2	2	5	29	4	0	0,02	0,02	0,05	0,37	0,04
XP_025105257.1	uncharacterized protein LOC112570831	56,499	0	0	0	165	188	139	0	0	0	5	5	3	0	0	0	0,35	0,35	0,2
XP_025081260.1	collagen alpha-1(I) chain-like	139,772	694	650	740	162	155	166	7	7	8	2	2	3	0,18	0,18	0,21	0,05	0,05	0,08
XP_025087575.1	metalloproteinase inhibitor 3-like isoform X3	18,289	0	0	0	161	0	143	0	0	0	2	0	2	0	0	0	0,43	0	0,43
XP_025081444.1	60S ribosomal protein L23	14,741	0	0	0	157	187	185	0	0	0	2	2	2	0	0	0	0,56	0,56	0,56
XP_025099638.1	ganglioside GM2 activator-like	22,163	313	330	294	156	81	107	3	3	3	3	3	3	0,56	0,56	0,56	0,57	0,56	0,56
XP_025096557.1	citrate synthase, mitochondrial-like	52,319	0	0	0	155	83	105	0	0	0	3	2	2	0	0	0	0,21	0,14	0,14
XP_025106401.1	uncharacterized protein LOC112571557 isoform X1	21,595	0	0	0	154	79	54	0	0	0	2	2	2	0	0	0	0,36	0,36	0,36
XP_025098389.1	uncharacterized protein LOC112566423	16,591	240	256	258	0	0	0	2	2	2	0	0	0	0,49	0,49	0,49	0	0	0
XP_025110318.1	12 kDa FK506-binding protein-like	12,476	0	0	0	149	103	112	0	0	0	5	3	4	0	0	0	2,69	1,19	1,84
XP_025081261.1	collagen alpha-1(I) chain-like isoform X1	134,816	558	558	649	149	168	195	3	3	2	2	2	3	0,08	0,08	0,05	0,05	0,05	0,08
XP_025104800.1	uncharacterized protein LOC112570531	72,519	395	474	388	145	158	170	5	6	6	3	4	4	0,32	0,39	0,39	0,15	0,2	0,2
XP_025104799.1	uncharacterized protein LOC112570529	73,828	527	616	510	138	136	220	2	2	6	2	3	3	0,44	0,51	0,51	0,1	0,15	0,15
XP_025076712.1	heterogeneous nuclear ribonucleoprotein 27C-like isoform X1	46,517	0	0	0	145	153	112	0	0	0	2	3	3	0	0	0	0,16	0,24	0,24

XP_025114047.1	uncharacterized protein LOC112576040	25,236	429	463	489	145	0	0	8	8	8	3	0	0	1,87	1,87	1,87	0,49	0	0
XP_025113673.1	fatty acid-binding protein, liver-like	15,04	189	174	189	143	111	108	5	5	3	3	3	3	1,98	1,98	0,92	0,93	0,92	0,92
XP_025081291.1	arsenite methyltransferase-like	39,189	0	0	0	139	128	116	0	0	0	5	3	4	0	0	0	0,53	0,29	0,41
XP_025082245.1	LQP: myosin heavy chain, non-muscle-like	231,417	0	0	0	138	105	116	0	0	0	2	2	2	0	0	0	0,03	0,03	0,03
XP_025098518.1	stefin-C-like	12,205	0	0	0	134	130	119	0	0	0	2	2	2	0	0	0	0,7	0,7	0,7
XP_025086814.1	uncharacterized protein LOC112559692	38,466	0	0	0	132	173	113	0	0	0	3	3	3	0	0	0	0,3	0,3	0,3
XP_025102335.1	uncharacterized protein LOC112568962 isoform X1	110,994	0	0	52	132	129	147	0	0	2	4	3	3	0	0	0,06	0,13	0,1	0,1
XP_025113277.1	40S ribosomal protein S18	17,782	0	0	0	131	201	155	0	0	0	5	6	6	0	0	0	1,53	2,04	2,04
XP_025086205.1	nucleoside diphosphate kinase-like	18,639	0	0	0	131	141	109	0	0	0	3	3	4	0	0	0	0,7	0,7	1,03
XP_025084055.1	uncharacterized protein LOC112558076	19,816	134	134	109	129	139	103	2	2	2	3	3	2	0,39	0,39	0,39	0,65	0,65	0,39
XP_025090775.1	LQP: tensin-1-like	181,636	1401	1428	1411	127	127	156	14	14	12	4	4	4	0,32	0,32	0,27	0,08	0,08	0,08
XP_025086204.1	40S ribosomal protein S3	27,597	0	0	0	127	106	135	0	0	0	4	3	5	0	0	0	0,62	0,44	0,83
XP_025083419.1	small cardioactive peptides-like isoform X1	19,262	597	551	568	126	143	99	5	5	5	2	2	3	1,35	1,35	1,35	0,41	0,41	0,67
XP_025107794.1	ras-related protein Rap1	21,046	0	0	0	126	125	157	0	0	0	2	2	2	0	0	0	0,37	0,37	0,37
XP_025093088.1	60S ribosomal protein L6-like	24,672	0	0	0	126	134	114	0	0	0	4	3	3	0	0	0	0,72	0,5	0,5
XP_025083894.1	profilin-like	14,445	0	0	0	125	112	115	0	0	0	4	3	3	0	0	0	1,47	0,97	0,97
XP_025083634.1	thioredoxin-1-like	11,743	265	255	262	124	139	125	4	5	4	2	2	3	2	4,2	2,95	0,73	0,73	1,28
XP_025094101.1	FK506-binding protein 2-like	24,869	404	399	339	121	94	99	6	6	6	3	3	4	1,55	1,55	1,23	0,71	0,49	0,71
XP_025088543.1	uncharacterized protein LOC112560733	22,537	0	0	0	119	0	81	0	0	0	2	0	2	0	0	0	0,34	0	0,34
XP_025111371.1	LQP: 40S ribosomal protein S12-like	15,206	265	209	202	118	120	87	6	6	6	3	3	3	2,63	2,63	2,63	0,91	0,91	0,91
XP_025081568.1	calcium-binding protein LPS1-beta-like	17,91	440	349	459	0	156	0	6	6	6	0	2	0	2,02	2,02	2,02	0	0,45	0
XP_025106310.1	gelsolin-like protein 2	42,15	0	0	0	118	0	100	0	0	0	2	0	2	0	0	0	0,17	0	0,17
XP_025112868.1	uncharacterized protein LOC112575321 isoform X1	270,028	91	77	0	116	135	130	2	2	0	5	5	4	0,03	0,03	0	0,06	0,06	0,05
XP_025086639.1	LQP: protein lethal(2)essential for life-like	25,522	0	52	0	113	123	91	0	2	0	3	4	2	0	0,48	0	0,48	0,68	0,3
XP_025092572.1	von Willebrand factor D and EGF domain-containing protein-like	129,551	126	116	98	113	0	172	2	2	2	2	0	4	0,05	0,05	0,05	0,05	0	0,11
XP_025091415.1	40S ribosomal protein S2	30,526	0	0	0	113	182	203	0	0	0	5	5	6	0	0	0	0,73	0,73	0,93
XP_025109048.1	peroxiredoxin-like isoform X1	26,496	168	126	64	112	112	69	3	3	2	3	3	2	0,46	0,65	0,28	0,46	0,46	0,28
XP_025108453.1	ubiquitin-like modifier-activating enzyme 1	118,163	0	0	0	110	157	134	0	0	0	3	6	4	0	0	0	0,09	0,19	0,12
XP_025111655.1	spliceosome RNA helicase DDX39B	49,265	0	0	0	109	94	52	0	0	0	4	4	2	0	0	0	0,31	0,31	0,15
XP_025105436.1	calpain-B-like isoform X1	93,005	0	0	0	108	133	91	0	0	0	2	3	2	0	0	0	0,08	0,12	0,08
XP_025085932.1	plastin-1-like	70,411	0	0	0	105	107	96	0	0	0	2	6	3	0	0	0	0,1	0,33	0,15
XP_025087916.1	40S ribosomal protein S3a	29,98	0	0	0	104	106	78	0	0	0	3	4	4	0	0	0	0,4	0,56	0,56
XP_025090773.1	60S ribosomal protein L23a-like isoform X1	20,864	0	0	0	104	109	69	0	0	0	2	2	2	0	0	0	0,37	0,37	0,37
XP_025114384.1	PDZ and LIM domain protein 3-like isoform X1	39,885	456	421	412	104	94	101	7	8	7	3	2	3	0,8	0,96	0,8	0,29	0,18	0,29
XP_025082063.1	tropomodulin-like	39,402	0	0	0	104	121	0	0	0	0	2	2	0	0	0	0	0,19	0,19	0
XP_025104850.1	elongation factor 1-gamma-like	50,033	0	0	0	103	83	0	0	0	0	3	2	0	0	0	0	0,22	0,14	0
XP_025107162.1	LQP: 60S ribosomal protein L3-like	46,565	0	0	0	103	212	197	0	0	0	2	4	4	0	0	0	0,16	0,33	0,33
XP_025109085.1	laminin subunit alpha-like	411,067	0	55	0	103	220	118	0	2	0	3	5	4	0	0,02	0	0,03	0,05	0,03
XP_025094847.1	glycine--tRNA ligase-like	88,188	0	0	0	101	125	134	0	0	0	2	3	3	0	0	0	0,08	0,12	0,12
XP_025088674.1	prosaposin-like	99,532	797	780	803	0	0	0	10	12	8	0	0	0	0,45	0,56	0,36	0	0	0
XP_025096773.1	transaldolase-like isoform X1	36,898	0	0	0	100	112	150	0	0	0	3	4	3	0	0	0	0,31	0,44	0,31
XP_025080657.1	uncharacterized protein LOC112556148	19,74	360	368	375	0	0	132	2	2	2	0	0	2	0,4	0,4	0,4	0	0	0,4
XP_025112782.1	60S ribosomal protein L5-like	36,194	0	0	0	99	0	96	0	0	0	3	0	2	0	0	0	0,32	0	0,2
XP_025077943.1	LQP: cartilage matrix protein-like	70,84	314	195	378	98	0	133	5	5	6	3	0	4	0,27	0,27	0,33	0,15	0	0,21
XP_025109560.1	LQP: 40S ribosomal protein S21-like	9,265	221	197	218	0	182	0	2	2	3	0	2	0	1	1	1,82	0	1	0
XP_025105802.1	lactoylglutathione lyase-like isoform X1	20,346	77	72	66	95	96	90	2	2	2	3	2	2	0,38	0,38	0,38	0,63	0,38	0,38
XP_025081510.1	pyruvate kinase PKM-like isoform X1	69,744	0	0	0	95	136	155	0	0	0	2	3	3	0	0	0	0,1	0,16	0,16
XP_025109667.1	polypyrimidine tract-binding protein 2-like isoform X1	64,175	0	0	0	94	150	0	0	0	0	2	2	0	0	0	0	0,11	0,11	0
XP_025088080.1	60S ribosomal protein L32-like	15,994	0	0	0	93	104	0	0	0	0	2	2	0	0	0	0	0,51	0,51	0
XP_025086455.1	dihydropyrimidinase-like	63,125	71	0	0	93	67	106	3	0	0	4	3	3	0,17	0	0	0,24	0,17	0,17
XP_025104144.1	mammalian ependymin-related protein 1-like	24,212	261	176	247	93	139	113	4	4	4	2	2	2	0,73	0,73	0,73	0,32	0,32	0,32
XP_025106938.1	mitochondrial-processing peptidase subunit beta-like	54,081	0	0	0	0	64	134	0	0	0	0	3	2	0	0	0	0	0,21	0,13

XP_025113992.1	quinone oxidoreductase-like isoform X1	35,93	0	0	0	0	177	101	0	0	0	0	3	3	0	0	0	0	0,32	0,32
XP_025085423.1	40S ribosomal protein S10-like	18,224	0	0	0	90	0	59	0	0	0	2	0	2	0	0	0	0,44	0	0,44
XP_025090121.1	60S acidic ribosomal protein P1-like	12,009	0	0	0	90	98	96	0	0	0	2	2	2	0	0	0	0,72	0,72	0,72
XP_025078139.1	60S ribosomal protein L17-like	21,728	0	0	0	90	61	60	0	0	0	3	2	3	0	0	0	0,58	0,36	0,58
XP_025087593.1	glycogen [starch] synthase-like	79,825	0	0	0	89	169	104	0	0	0	3	3	2	0	0	0	0,14	0,14	0,09
XP_025092427.1	flotillin-2-like	46,782	0	0	0	89	70	69	0	0	0	3	2	3	0	0	0	0,24	0,15	0,24
XP_025080098.1	60S ribosomal protein L8-like	28,685	0	0	0	89	115	82	0	0	0	3	3	2	0	0	0	0,42	0,42	0,26
XP_025105813.1	ribosome-binding protein 1-like isoform X1	149,175	0	0	0	0	135	152	0	0	0	0	3	4	0	0	0	0	0,07	0,1
XP_025081296.1	proteasome subunit alpha type-2	26,349	0	0	0	88	108	87	0	0	0	2	2	2	0	0	0	0,29	0,29	0,29
XP_025076135.1	fructose-1,6-bisphosphatase 1-like	37,452	115	103	0	87	130	54	3	2	0	3	4	2	0,31	0,2	0	0,31	0,43	0,2
XP_025082862.1	PDZ and LIM domain protein 5-like	22,253	674	762	770	86	126	75	7	8	7	2	2	2	1,84	2,3	1,84	0,35	0,35	0,35
XP_025082818.1	triosephosphate isomerase-like	28,702	83	0	105	86	115	110	3	0	3	4	4	3	0,42	0	0,42	0,59	0,59	0,42
XP_025092955.1	aconitate hydratase, mitochondrial-like	85,379	0	0	0	86	172	88	0	0	0	2	3	2	0	0	0	0,08	0,13	0,08
XP_025116005.1	uncharacterized protein LOC112577210 isoform X1	36,719	79	74	109	85	83	62	4	4	3	3	2	2	0,44	0,44	0,31	0,31	0,2	0,2
XP_025103394.1	probable methylmalonate-semialdehyde dehydrogenase [acylating], mitochondrial	57,119	0	0	0	0	105	176	0	0	0	0	2	3	0	0	0	0	0,13	0,19
XP_025100959.1	uncharacterized protein LOC112568087	21,521	0	0	0	85	58	0	0	0	0	3	2	0	0	0	0	0,59	0,36	0
XP_025112729.1	uncharacterized protein LOC112575240	33,74	0	0	0	84	74	74	0	0	0	2	2	2	0	0	0	0,22	0,22	0,22
XP_025107611.1	laminin subunit beta-1-like	202,317	0	0	0	84	69	59	0	0	0	4	2	2	0	0	0	0,07	0,03	0,03
XP_025103993.1	titin homolog isoform X1	222,758	0	2200	0	83	106	0	0	35	0	7	4	0	0	0,7	0	0,11	0,06	0
XP_025089133.1	fatty acid-binding protein, adipocyte-like	14,871	312	305	243	81	107	80	3	3	3	2	2	2	0,93	0,93	0,93	0,55	0,55	0,55
XP_025087837.1	60S ribosomal protein L12-like	17,801	0	0	0	81	98	120	0	0	0	2	3	4	0	0	0	0,45	0,74	1,1
XP_025116283.1	uncharacterized protein LOC112577430 isoform X1	72,441	0	0	0	80	126	65	0	0	0	4	4	3	0	0	0	0,21	0,2	0,15
XP_025096378.1	ras-like GTP-binding protein RHO	36,715	0	0	0	79	56	51	0	0	0	3	3	2	0	0	0	0,31	0,31	0,2
XP_025091729.1	60S ribosomal protein L19-like	23,397	0	0	0	77	0	42	0	0	0	2	0	2	0	0	0	0,33	0	0,33
XP_025096000.1	60S ribosomal protein L27-like	16,229	0	0	0	77	61	77	0	0	0	3	3	3	0	0	0	0,84	0,83	0,83
XP_025097402.1	tubulin polymerization-promoting protein family member 2-like	21,142	105	82	0	0	0	0	2	2	0	0	0	0	0,37	0,37	0	0	0	0
XP_025112013.1	40S ribosomal protein S9	22,39	0	0	0	76	73	72	0	0	0	2	3	2	0	0	0	0,34	0,56	0,34
XP_025094074.1	LQP: uncharacterized protein LOC112563873	128,795	0	0	0	74	66	38	0	0	0	2	3	2	0	0	0	0,05	0,08	0,05
XP_025092020.1	60S ribosomal protein L7-like	28,447	0	0	0	72	89	82	0	0	0	3	4	3	0	0	0	0,42	0,6	0,42
XP_025087859.1	LQP: BTB/POZ domain-containing protein KCTD12-like	30,712	76	0	0	71	90	81	2	0	0	2	2	2	0,24	0	0	0,24	0,24	0,24
XP_025103882.1	histone H2A	13,649	0	0	0	70	114	81	0	0	0	3	4	4	0	0	0	1,05	1,6	1,6
XP_025111693.1	collagen alpha-1(I) chain-like	171,566	293	315	242	0	0	0	5	5	5	0	0	0	0,13	0,13	0,13	0	0	0
XP_025098586.1	cystatin-B-like	11,319	0	51	54	0	0	0	0	2	2	0	0	0	0	0,77	0,77	0	0	0
XP_025098510.1	40S ribosomal protein S15Aa	14,976	0	0	0	69	87	0	0	0	0	2	2	0	0	0	0	0,55	0,55	0
XP_025110976.1	phosphoglucomutase-1-like	62,002	0	0	0	69	74	0	0	0	0	2	2	0	0	0	0	0,11	0,11	0
XP_025097167.1	60S ribosomal protein L24-like	17,809	0	0	0	68	85	59	0	0	0	2	2	2	0	0	0	0,45	0,45	0,45
XP_025092850.1	dolichyl-diphosphooligosaccharide--protein glycosyltransferase subunit 2-like	70,075	0	0	0	68	0	57	0	0	0	2	0	2	0	0	0	0,1	0	0,1
XP_025115104.1	hemocyte protein-glutamine gamma-glutamyltransferase-like isoform X1	87,766	0	0	0	66	65	65	0	0	0	4	4	3	0	0	0	0,17	0,17	0,12
XP_025107641.1	universal stress protein A-like protein	17,455	0	0	0	66	0	75	0	0	0	2	0	2	0	0	0	0,46	0	0,46
XP_025087122.1	myotrophin-like	13,092	345	413	338	63	121	67	5	4	5	3	3	3	2,46	1,7	2,46	1,11	1,1	1,1
XP_025105795.1	protein phosphatase 1B-like isoform X1	43,227	0	0	0	63	65	59	0	0	0	3	3	3	0	0	0	0,26	0,26	0,26
XP_025093952.1	uncharacterized protein LOC112563809	28,586	0	0	0	63	65	78	0	0	0	4	5	3	0	0	0	0,59	0,79	0,42
XP_025097674.1	peroxiredoxin-2-like	28,899	0	73	0	63	74	82	0	2	0	2	2	2	0	0,41	0	0,26	0,26	0,26
XP_025088440.1	uncharacterized protein LOC112560658 isoform X1	15,836	355	341	381	0	0	0	2	2	2	0	0	0	0,51	0,51	0,51	0	0	0
XP_025101599.1	phospholipid transfer protein C2CD2L-like	81,23	248	117	160	0	88	74	3	3	3	0	2	2	0,13	0,13	0,13	0	0,09	0,09
XP_025081438.1	LIM and SH3 domain protein F42H10.3-like isoform X1	35,63	0	0	0	60	69	70	0	0	0	2	3	2	0	0	0	0,21	0,33	0,21
XP_025086082.1	LQP: septin-11-like	47,525	0	0	0	59	70	31	0	0	0	2	2	2	0	0	0	0,15	0,15	0,15
XP_025083868.1	heterogeneous nuclear ribonucleoprotein D-like	38,546	0	0	0	56	78	0	0	0	0	2	2	0	0	0	0	0,19	0,19	0
XP_025109837.1	2-iminobutanoate/2-iminopropanoate deaminase-like	14,184	406	404	358	0	0	0	6	6	6	0	0	0	2,96	2,96	2,96	0	0	0
XP_025100558.1	40S ribosomal protein S19-like isoform X1	20,188	0	0	0	54	75	0	0	0	0	2	2	0	0	0	0	0,39	0,39	0
XP_025097427.1	beta-parvin-like	42,271	0	0	0	54	49	43	0	0	0	2	2	2	0	0	0	0,17	0,17	0,17
XP_025106346.1	dolichyl-diphosphooligosaccharide--protein glycosyltransferase subunit 1-like	68,993	0	0	0	0	57	43	0	0	0	0	3	2	0	0	0	0	0,16	0,1

XP_025090868.1	glutathione peroxidase-like	26,603	91	75	98	49	0	0	4	4	4	2	0	0	0,65	0,65	0,65	0,28	0	0
XP_025095897.1	fucose mutarotase-like	16,766	239	350	305	0	0	67	5	5	5	0	0	2	1,66	1,66	2,24	0	0	0,48
XP_025095580.1	chitotriosidase-1-like isoform X1	38,127	0	0	0	47	58	43	0	0	0	3	2	2	0	0	0	0,3	0,19	0,19
XP_025078843.1	glycogenin-1-like isoform X1	66,174	256	235	245	47	42	54	6	6	7	3	3	3	0,36	0,36	0,43	0,17	0,17	0,17
XP_025087561.1	uncharacterized protein LOC112560159 isoform X1	15,686	205	173	198	0	0	0	2	2	2	0	0	0	0,52	0,52	0,52	0	0	0
XP_025106405.1	dystroglycan-like	95,017	357	339	301	0	0	0	4	4	3	0	0	0	0,15	0,15	0,11	0	0	0
XP_025086042.1	Na(+)/H(+) exchange regulatory cofactor NHE-RF1-like isoform X1	53,7	289	292	278	0	0	0	5	7	8	0	0	0	0,37	0,55	0,65	0	0	0
XP_025077177.1	60S ribosomal protein L4-like	45,469	0	0	0	43	32	0	0	0	0	2	2	0	0	0	0	0,16	0,16	0
XP_025085605.1	annexin A7-like	56,197	0	0	0	0	72	92	0	0	0	0	3	4	0	0	0	0	0,2	0,27
XP_025079223.1	uncharacterized protein LOC112555178	46,253	200	137	183	0	0	0	4	4	4	0	0	0	0,34	0,34	0,34	0	0	0
XP_025107147.1	transforming growth factor-beta-induced protein ig-h3-like	34,438	896	891	848	0	55	0	10	12	11	0	2	0	1,91	2,54	2,21	0	0,21	0
XP_025079302.1	uncharacterized protein LOC112555220	12,065	162	154	175	0	0	0	4	4	4	0	0	0	1,92	1,92	1,92	0	0	0
XP_025092851.1	tumor protein D54-like isoform X1	30,235	132	0	90	0	0	0	2	0	2	0	0	0	0,25	0	0,25	0	0	0
XP_025100206.1	ATP synthase-coupling factor 6, mitochondrial-like	15,27	284	264	229	0	31	0	5	6	6	0	2	0	1,91	2,6	2,6	0	0,53	0
XP_025090122.1	60S ribosomal protein L30-like	13,181	0	0	0	38	0	35	0	0	0	2	0	2	0	0	0	0,64	0	0,64
XP_025076144.1	beta-1,3-glucan-binding protein-like	50,673	0	0	0	0	44	97	0	0	0	0	3	3	0	0	0	0	0,22	0,22
XP_025078861.1	pollen-specific leucine-rich repeat extensin-like protein 1	47,92	0	171	84	0	0	0	0	2	2	0	0	0	0	0,15	0,15	0	0	0
XP_025097582.1	cathepsin L1-like	40,372	403	322	313	34	0	0	5	5	5	2	0	0	0,51	0,51	0,51	0,18	0	0
XP_025088165.1	histone H3.v1-like	12,893	122	0	73	0	0	50	2	0	2	0	0	2	0,66	0	0,66	0	0	0,66
XP_025104532.1	short-chain collagen C4-like	32,968	242	149	156	0	0	0	6	5	5	0	0	0	0,84	0,66	0,66	0	0	0
XP_025093470.1	dynactin subunit 2-like	45,625	178	109	99	0	0	0	4	3	4	0	0	0	0,34	0,25	0,34	0	0	0
XP_025113923.1	fatty acid-binding protein, liver-like	15,22	213	192	163	0	0	0	4	4	4	0	0	0	1,36	1,36	1,36	0	0	0
XP_025112876.1	uncharacterized protein LOC112575323	98,079	0	0	0	0	79	33	0	0	0	0	2	2	0	0	0	0	0,07	0,07
XP_025114330.1	tubulin beta chain	50,319	559	528	447	0	0	0	11	10	9	0	0	0	1,09	0,95	0,83	0	0	0
XP_025108539.1	ankyrin-2-like isoform X1	361,409	0	0	0	0	243	140	0	0	0	0	5	3	0	0	0	0	0,05	0,03
XP_025081749.1	guanine nucleotide-binding protein subunit beta isoform X2	37,965	0	0	0	0	120	151	0	0	0	0	5	5	0	0	0	0	0,7	0,55
XP_025083230.1	LQP: melanotransferrin-like	80,302	234	147	0	0	0	0	3	2	0	0	0	0	0,13	0,09	0	0	0	0
XP_025082468.1	LQP: 60S ribosomal protein L18-like	21,639	0	0	0	0	72	53	0	0	0	0	2	2	0	0	0	0	0,36	0,36
XP_025093782.1	chitotriosidase-1-like	41,703	86	91	150	0	0	0	2	4	3	0	0	0	0,17	0,38	0,27	0	0	0
XP_025096536.1	ubiquitin-conjugating enzyme E2 2	20,496	0	0	0	0	56	30	0	0	0	0	2	2	0	0	0	0	0,38	0,38
XP_025079812.1	uncharacterized protein LOC112555596	17,644	269	286	203	0	0	0	3	3	3	0	0	0	0,75	0,75	0,75	0	0	0
XP_025082010.1	myosin-2 essential light chain-like	16,923	794	725	700	0	49	77	10	8	8	0	3	2	7,5	4,76	3,74	0	0,79	0,48
XP_025092057.1	thioredoxin domain-containing protein 17-like	14,159	57	54	72	0	0	0	2	3	2	0	0	0	0,58	0,99	0,58	0	0	0
XP_025095124.1	calmodulin-like	17,104	180	286	257	0	0	0	4	5	4	0	0	0	1,16	1,61	1,16	0	0	0
XP_025089653.1	leupaxin-like isoform X1	70,025	149	151	111	0	0	0	2	3	3	0	0	0	0,1	0,16	0,16	0	0	0
XP_025088872.1	complement component 1 Q subcomponent-binding protein, mitochondrial-like	31,24	0	57	81	0	0	0	0	2	2	0	0	0	0	0,24	0,24	0	0	0
XP_025104008.1	titin homolog isoform X3	207,977	1812	0	1957	0	0	100	30	0	25	0	0	4	0,63	0	0,5	0	0	0,07
XP_025111818.1	LQP: lysosomal alpha-mannosidase-like	113,194	122	99	135	0	0	0	3	2	2	0	0	0	0,09	0,06	0,06	0	0	0
XP_025083732.1	glycine, glutamate and proline-rich protein-like	23,715	326	192	121	0	0	0	3	2	3	0	0	0	0,52	0,32	0,52	0	0	0
XP_025094607.1	uncharacterized protein LOC112564180	43,433	197	214	129	0	0	0	3	4	3	0	0	0	0,26	0,36	0,26	0	0	0
XP_025090869.1	ATPase inhibitor mai-2, mitochondrial-like isoform X1	12,938	155	124	65	0	0	0	4	5	3	0	0	0	1,72	2,49	1,12	0	0	0
XP_025082604.1	basement membrane-specific heparan sulfate proteoglycan core protein-like isoform X5	524,897	4660	4468	0	0	0	0	81	75	0	0	0	0	0,69	0,64	0	0	0	0
XP_025081479.1	talin-1-like isoform X13	235,752	1456	1357	1405	0	0	0	24	23	21	0	0	0	0,41	0,39	0,35	0	0	0
XP_025092885.1	proprotein convertase subtilisin/kexin type 5-like	17,517	914	848	779	0	0	0	2	2	2	0	0	0	0,75	0,75	0,75	0	0	0
XP_025091493.1	extensin-like isoform X7	103,251	838	1056	842	0	0	0	15	15	15	0	0	0	0,58	0,58	0,58	0	0	0
XP_025114843.1	uncharacterized protein LOC112576527 isoform X11	36,026	702	834	0	0	0	0	6	5	0	0	0	0	1,3	1,1	0	0	0	0
XP_025093891.1	LQP: tubulin alpha-1A chain-like	50,691	686	552	432	0	0	0	3	2	2	0	0	0	0,7	0,7	0,59	0	0	0
XP_025091013.1	collagen alpha-5(VI) chain-like isoform X1	51,217	602	0	614	0	0	0	10	0	8	0	0	0	0,93	0	0,69	0	0	0
XP_025100362.1	calmodulin, striated muscle-like isoform X2	11,862	566	553	0	0	0	0	6	6	0	0	0	0	5,73	5,73	0	0	0	0
XP_025105683.1	uncharacterized protein LOC112571088	74,251	556	645	0	0	0	0	3	3	0	0	0	0	0,44	0,51	0	0	0	0
XP_025079883.1	collagen alpha-2(IV) chain-like	139,15	453	311	332	0	0	0	6	6	5	0	0	0	0,19	0,16	0,13	0	0	0
XP_025081440.1	LIM and SH3 domain protein F42H10.3-like isoform X3	34,807	418	429	433	0	0	0	7	6	7	0	0	0	0,96	0,78	0,96	0	0	0

XP_025079333.1	calmodulin-like	16,677	340	336	280	0	0	0	3	3	3	0	0	0	0,81	0,81	0,81	0	0	0
XP_025107571.1	inter-alpha-trypsin inhibitor heavy chain H3-like	99,962	329	413	357	0	0	0	11	12	11	0	0	0	0,45	0,5	0,45	0	0	0
XP_025106287.1	dipeptidyl peptidase 1-like	52,995	311	357	331	0	0	0	4	4	4	0	0	0	0,29	0,29	0,29	0	0	0
XP_025079817.1	LQP: uncharacterized protein LOC112555602	69,351	309	335	278	0	0	0	5	5	7	0	0	0	0,28	0,28	0,41	0	0	0
XP_025116102.1	calbindin-32-like isoform X1	36,511	305	402	363	0	0	0	5	6	6	0	0	0	0,58	0,73	0,73	0	0	0
XP_025079882.1	collagen alpha-5(IV) chain-like	173,514	301	270	185	0	0	0	6	6	6	0	0	0	0,12	0,12	0,12	0	0	0
XP_025106308.1	gelsolin-like protein 2 isoform X1	41,888	297	249	229	0	0	0	5	5	5	0	0	0	0,49	0,49	0,49	0	0	0
XP_025110417.1	reticulocalbin-2-like	35,983	284	274	236	0	0	0	6	7	6	0	0	0	0,75	0,92	0,75	0	0	0
XP_025080910.1	lipoma-preferred partner homolog	31,112	279	175	260	0	0	0	5	5	4	0	0	0	0,71	0,71	0,54	0	0	0
XP_025092566.1	protein obstructor-E-like	18,955	270	297	304	0	0	0	5	5	5	0	0	0	1,83	1,38	1,38	0	0	0
XP_025086087.1	uncharacterized protein LOC112559237 isoform X1	27,276	267	246	205	0	0	0	3	3	2	0	0	0	0,44	0,44	0,28	0	0	0
XP_025101009.1	uncharacterized protein LOC112568120 isoform X1	133,416	240	194	228	0	0	0	5	5	5	0	0	0	0,14	0,14	0,14	0	0	0
XP_025096935.1	whey acidic protein-like isoform X1	23,165	226	252	141	0	0	0	2	3	3	0	0	0	0,33	0,54	0,54	0	0	0
XP_025113591.1	uncharacterized protein LOC112575783	25,007	216	205	195	0	0	0	3	3	3	0	0	0	0,49	0,49	0,49	0	0	0
XP_025110822.1	glucosidase 2 subunit beta-like isoform X1	64,125	211	189	105	0	0	0	7	5	4	0	0	0	0,44	0,3	0,23	0	0	0
XP_025111425.1	nuclear autoantigenic sperm protein-like	78,609	186	176	133	0	0	0	5	3	4	0	0	0	0,24	0,14	0,19	0	0	0
XP_025099450.1	temptin-like isoform X1	16,743	184	171	183	0	0	0	2	2	2	0	0	0	0,48	0,48	0,48	0	0	0
XP_025113972.1	uncharacterized protein LOC112575997	15,844	178	159	92	0	0	0	2	2	2	0	0	0	0,51	0,51	0,51	0	0	0
XP_025098500.1	uncharacterized protein LOC112566496	11,776	173	188	203	0	0	0	2	2	2	0	0	0	0,73	0,73	0,73	0	0	0
XP_025099452.1	temptin-like isoform X3	16,563	171	110	0	0	0	0	2	2	0	0	0	0	0,49	0,49	0	0	0	0
XP_025080338.1	uncharacterized protein LOC112555941	14,457	167	217	185	0	0	0	3	4	3	0	0	0	0,97	1,47	0,97	0	0	0
XP_025079520.1	LAMP family protein lmp-1-like	39,793	163	215	111	0	0	0	2	2	2	0	0	0	0,18	0,18	0,18	0	0	0
XP_025108426.1	low-density lipoprotein receptor class A domain-containing protein 3-like	17,586	154	166	155	0	0	0	2	2	2	0	0	0	0,45	0,45	0,45	0	0	0
XP_025096824.1	SH3 domain-binding glutamic acid-rich protein homolog	33,417	154	128	92	0	0	0	4	4	4	0	0	0	0,49	0,49	0,49	0	0	0
XP_025114909.1	programmed cell death protein 5-like	15,134	150	236	92	0	0	0	3	3	4	0	0	0	0,91	0,91	1,38	0	0	0
XP_025099231.1	uncharacterized protein LOC112567014	13,109	149	156	120	0	0	0	2	3	3	0	0	0	0,64	1,1	1,1	0	0	0
XP_025077656.1	cytochrome b-c1 complex subunit 6, mitochondrial-like	12,11	146	103	0	0	0	0	2	2	0	0	0	0	0,71	0,71	0	0	0	0
XP_025108739.1	uncharacterized protein LOC112572951 isoform X1	84,553	138	146	92	0	0	0	2	2	3	0	0	0	0,08	0,08	0,13	0	0	0
XP_025099105.1	uncharacterized protein LOC112566898 isoform X1	43,391	137	92	89	0	0	0	2	2	2	0	0	0	0,17	0,17	0,17	0	0	0
XP_025088922.1	chitin-binding domain protein cbd-1-like	17,671	133	79	99	0	0	0	3	3	3	0	0	0	0,75	0,75	0,75	0	0	0
XP_025081201.1	ATP synthase subunit delta, mitochondrial-like	17,628	132	152	0	0	0	0	3	3	0	0	0	0	0,75	0,75	0	0	0	0
XP_025087541.1	myomodulin neuropeptides 1-like	37,555	130	126	127	0	0	0	2	2	2	0	0	0	0,2	0,2	0,2	0	0	0
XP_025091642.1	C-type lectin domain family 4 member F-like	78,968	108	111	0	0	0	0	2	2	0	0	0	0	0,14	0,14	0	0	0	0
XP_025081096.1	uncharacterized protein LOC112556364 isoform X1	41,668	121	129	132	0	0	0	2	2	2	0	0	0	0,17	0,17	0,17	0	0	0
XP_025085233.1	jupiter microtubule associated homolog 1-like	15,8	117	122	115	0	0	0	3	3	3	0	0	0	0,86	0,86	0,86	0	0	0
XP_025095496.1	thioredoxin, mitochondrial-like	18,684	110	110	0	0	0	0	2	2	0	0	0	0	0,42	0,42	0	0	0	0
XP_025080183.1	C-type mannose receptor 2-like	39,263	110	0	108	0	0	0	2	0	2	0	0	0	0,19	0	0,19	0	0	0
XP_025083875.1	perlucin-like	17,84	109	132	0	0	0	0	2	2	0	0	0	0	0,44	0,44	0	0	0	0
XP_025108114.1	sortilin-related receptor-like	33,215	0	136	137	0	0	0	0	2	2	0	0	0	0	0,22	0,22	0	0	0
XP_025091465.1	cathepsin B-like	37,094	107	136	93	0	0	0	3	3	2	0	0	0	0,31	0,31	0,2	0	0	0
XP_025077166.1	ubiquilin-1-like isoform X1	61,23	106	107	0	0	0	0	2	3	0	0	0	0	0,12	0,18	0	0	0	0
XP_025091949.1	uncharacterized protein LOC112562719 isoform X1	228,702	104	85	74	0	0	0	2	2	2	0	0	0	0,03	0,03	0,03	0	0	0
XP_025110414.1	UV excision repair protein RAD23 homolog B-like	42,339	102	138	79	0	0	0	3	3	4	0	0	0	0,27	0,27	0,37	0	0	0
XP_025115296.1	nucleobindin-2-like isoform X1	62,651	101	0	49	0	0	0	2	0	2	0	0	0	0,11	0	0,11	0	0	0
XP_025091078.1	calcium-regulated heat stable protein 1-like	16,322	99	96	68	0	0	0	3	2	2	0	0	0	0,83	0,49	0,49	0	0	0
XP_025097352.1	LQP: src substrate cortactin-like	61,172	97	148	142	0	0	0	2	3	3	0	0	0	0,12	0,18	0,18	0	0	0
XP_025102573.1	very low-density lipoprotein receptor-like	17,855	97	78	90	0	0	0	2	2	2	0	0	0	0,44	0,74	0,74	0	0	0
XP_025113971.1	uncharacterized protein LOC112575996 isoform X2	18,111	92	107	0	0	0	0	2	2	0	0	0	0	0,44	0,44	0	0	0	0
XP_025088678.1	uncharacterized protein LOC112560828	23,978	85	160	171	0	0	0	2	2	2	0	0	0	0,32	0,32	0,32	0	0	0
XP_025094190.1	death-associated protein 1-like	12,171	85	47	46	0	0	0	3	2	3	0	0	0	1,22	0,7	1,22	0	0	0
XP_025106781.1	voltage-dependent calcium channel subunit alpha-2/delta-2-like isoform X1	133,699	85	64	0	0	0	0	2	2	0	0	0	0	0,05	0,05	0	0	0	0
XP_025096696.1	insulin-like growth factor-binding protein-related protein 1	26,82	84	37	45	0	0	0	2	2	2	0	0	0	0,28	0,28	0,28	0	0	0

XP_025094826.1	acyl-CoA-binding protein-like	9,701	71	82	0	0	0	0	2	2	0	0	0	0	0,94	0,94	0	0	0	0
XP_025082802.1	formin-like protein 3	26,939	70	0	53	0	0	0	2	0	2	0	0	0	0,28	0	0,28	0	0	0
XP_025105588.1	endoglucanase-like	21,807	67	58	78	0	0	0	2	2	3	0	0	0	0,58	0,58	0,84	0	0	0
XP_025089735.1	proteasomal ubiquitin receptor ADRM1-like isoform X1	45,778	65	94	89	0	0	0	2	2	2	0	0	0	0,16	0,16	0,16	0	0	0
XP_025114839.1	uncharacterized protein LOC112576500 isoform X8	158,195	53	76	78	0	0	0	3	4	4	0	0	0	0,07	0,09	0,09	0	0	0
XP_025077341.1	tubulin-specific chaperone A-like	12,748	51	0	45	0	0	0	2	0	2	0	0	0	0,66	0	0,66	0	0	0
XP_025079545.1	uncharacterized protein LOC112555390	35,749	50	56	57	0	0	0	2	2	2	0	0	0	0,21	0,21	0,21	0	0	0
XP_025092033.1	uncharacterized protein LOC112562770 isoform X1	68,866	46	61	69	0	0	0	3	2	3	0	0	0	0,16	0,1	0,16	0	0	0
XP_025096197.1	uncharacterized protein LOC112565114	13,661	39	68	50	0	0	0	2	2	2	0	0	0	0,61	0,61	0,61	0	0	0
XP_025082044.1	calexcitin-2-like	21,865	0	82	67	0	0	0	0	3	2	0	0	0	0	0,57	0,35	0	0	0
XP_025108516.1	uncharacterized protein LOC112572830	35,046	0	80	131	0	0	0	0	2	3	0	0	0	0	0,21	0,33	0	0	0
