# Supplementary material for: Pomacea canaliculata Ampullar Proteome: A Nematode-Based Bio-Pesticide Induces Changes in Metabolic and Stress-Related Pathways
Source: Biology (Basel). 2021 Oct 15;10(10):1049. doi: 10.3390/biology10101049 (PMC8533556; doi:10.3390/biology10101049)
Supplement: Supplementary file 1 [file biology-10-01049-s001.zip › Table S2.pdf]

Table S2. List of proteins exclusively identified in either AmpC or AmpN samples.

Exclusively found in AmpC	
**Accession**	**Description**
XP_025077978.1	filamin-A-like isoform X3
XP_025095035.1	myosin regulatory light chain LC-2, mantle muscle-like isoform X2
XP_025096925.1	alpha-crystallin B chain-like
XP_025087032.1	uncharacterized protein LOC112559814 isoform X3
XP_025087033.1	thymosin beta-like isoform X4
XP_025098389.1	uncharacterized protein LOC112566423
XP_025114047.1	uncharacterized protein LOC112576040
XP_025081568.1	calcium-binding protein LPS1-beta-like
XP_025088674.1	prosaposin-like
XP_025080657.1	uncharacterized protein LOC112556148
XP_025109560.1	LQP: 40S ribosomal protein S21-like
XP_025097402.1	tubulin polymerization-promoting protein family member 2-like
XP_025111693.1	collagen alpha-1(I) chain-like
XP_025098586.1	cystatin-B-like
XP_025088440.1	uncharacterized protein LOC112560658 isoform X1
XP_025109837.1	2-iminobutanoate/2-iminopropanoate deaminase-like
XP_025090868.1	glutathione peroxidase-like
XP_025095897.1	fucose mutarotase-like
XP_025087561.1	uncharacterized protein LOC112560159 isoform X1
XP_025106405.1	dystroglycan-like
XP_025086042.1	Na(+)/H(+) exchange regulatory cofactor NHE-RF1-like isoform X1
XP_025079223.1	uncharacterized protein LOC112555178
XP_025107147.1	transforming growth factor-beta-induced protein ig-h3-like
XP_025079302.1	uncharacterized protein LOC112555220
XP_025092851.1	tumor protein D54-like isoform X1
XP_025100206.1	ATP synthase-coupling factor 6, mitochondrial-like
XP_025078861.1	pollen-specific leucine-rich repeat extensin-like protein 1
XP_025097582.1	cathepsin L1-like
XP_025088165.1	histone H3.v1-like
XP_025104532.1	short-chain collagen C4-like
XP_025093470.1	dynactin subunit 2-like
XP_025113923.1	fatty acid-binding protein, liver-like
XP_025114330.1	tubulin beta chain
XP_025083230.1	LQP: melanotransferrin-like
XP_025093782.1	chitotriosidase-1-like
XP_025079812.1	uncharacterized protein LOC112555596
XP_025092057.1	thioredoxin domain-containing protein 17-like
XP_025095124.1	calmodulin-like
XP_025089653.1	leupaxin-like isoform X1
XP_025088872.1	complement component 1 Q subcomponent-binding protein, mitochondrial-like
XP_025104008.1	titin homolog isoform X3
XP_025111818.1	LQP: lysosomal alpha-mannosidase-like
XP_025083732.1	glycine, glutamate and proline-rich protein-like

XP_025094607.1	uncharacterized protein LOC112564180
XP_025090869.1	ATPase inhibitor mai-2, mitochondrial-like isoform X1
XP_025082604.1	basement membrane-specific heparan sulfate proteoglycan core protein-like isoform X5
XP_025081479.1	talin-1-like isoform X13
XP_025092885.1	proprotein convertase subtilisin/kexin type 5-like
XP_025091493.1	extensin-like isoform X7
XP_025114843.1	uncharacterized protein LOC112576527 isoform X11
XP_025093891.1	LQP: tubulin alpha-1A chain-like
XP_025091013.1	collagen alpha-5(VI) chain-like isoform X1
XP_025100362.1	calmodulin, striated muscle-like isoform X2
XP_025105683.1	uncharacterized protein LOC112571088
XP_025079883.1	collagen alpha-2(IV) chain-like
XP_025081440.1	LIM and SH3 domain protein F42H10.3-like isoform X3
XP_025079333.1	calmodulin-like
XP_025107571.1	inter-alpha-trypsin inhibitor heavy chain H3-like
XP_025106287.1	dipeptidyl peptidase 1-like
XP_025079817.1	LQP: uncharacterized protein LOC112555602
XP_025116102.1	calbindin-32-like isoform X1
XP_025079882.1	collagen alpha-5(IV) chain-like
XP_025106308.1	gelsolin-like protein 2 isoform X1
XP_025110417.1	reticulocalbin-2-like
XP_025080910.1	lipoma-preferred partner homolog
XP_025092566.1	protein obstructor-E-like
XP_025086087.1	uncharacterized protein LOC112559237 isoform X1
XP_025101009.1	uncharacterized protein LOC112568120 isoform X1
XP_025096935.1	whey acidic protein-like isoform X1
XP_025113591.1	uncharacterized protein LOC112575783
XP_025110822.1	glucosidase 2 subunit beta-like isoform X1
XP_025111425.1	nuclear autoantigenic sperm protein-like
XP_025099450.1	temptin-like isoform X1
XP_025113972.1	uncharacterized protein LOC112575997
XP_025098500.1	uncharacterized protein LOC112566496
XP_025099452.1	temptin-like isoform X3
XP_025080338.1	uncharacterized protein LOC112555941
XP_025079520.1	LAMP family protein lmp-1-like
XP_025108426.1	low-density lipoprotein receptor class A domain-containing protein 3-like
XP_025096824.1	SH3 domain-binding glutamic acid-rich protein homolog
XP_025114909.1	programmed cell death protein 5-like
XP_025099231.1	uncharacterized protein LOC112567014
XP_025077656.1	cytochrome b-c1 complex subunit 6, mitochondrial-like
XP_025108739.1	uncharacterized protein LOC112572951 isoform X1
XP_025099105.1	uncharacterized protein LOC112566898 isoform X1
XP_025088922.1	chitin-binding domain protein cbd-1-like
XP_025081201.1	ATP synthase subunit delta, mitochondrial-like
XP_025087541.1	myomodulin neuropeptides 1-like
XP_025091642.1	C-type lectin domain family 4 member F-like
XP_025081096.1	uncharacterized protein LOC112556364 isoform X1

XP_025085233.1	jupiter microtubule associated homolog 1-like
XP_025095496.1	thioredoxin, mitochondrial-like
XP_025080183.1	C-type mannose receptor 2-like
XP_025083875.1	perlucin-like
XP_025108114.1	sortilin-related receptor-like
XP_025091465.1	cathepsin B-like
XP_025077166.1	ubiquilin-1-like isoform X1
XP_025091949.1	uncharacterized protein LOC112562719 isoform X1
XP_025110414.1	UV excision repair protein RAD23 homolog B-like
XP_025115296.1	nucleobindin-2-like isoform X1
XP_025091078.1	calcium-regulated heat stable protein 1-like
XP_025097352.1	LQP: src substrate cortactin-like
XP_025102573.1	very low-density lipoprotein receptor-like
XP_025113971.1	uncharacterized protein LOC112575996 isoform X2
XP_025088678.1	uncharacterized protein LOC112560828
XP_025094190.1	death-associated protein 1-like
XP_025106781.1	voltage-dependent calcium channel subunit alpha-2/delta-2-like isoform X1
XP_025096696.1	insulin-like growth factor-binding protein-related protein 1
XP_025094826.1	acyl-CoA-binding protein-like
XP_025082802.1	formin-like protein 3
XP_025105588.1	endoglucanase-like
XP_025089735.1	proteasomal ubiquitin receptor ADRM1-like isoform X1
XP_025114839.1	uncharacterized protein LOC112576500 isoform X8
XP_025077341.1	tubulin-specific chaperone A-like
XP_025079545.1	uncharacterized protein LOC112555390
XP_025092033.1	uncharacterized protein LOC112562770 isoform X1
XP_025096197.1	uncharacterized protein LOC112565114
XP_025082044.1	calexcitin-2-like
XP_025108516.1	uncharacterized protein LOC112572830
**Exclusively found in AmpN**	
**Accession**	**Description**
XP_025115938.1	tubulin beta-4B chain-like
XP_025109819.1	tubulin alpha-2/alpha-4 chain
XP_025086180.1	tubulin alpha-1A chain-like
XP_025093240.1	tubulin alpha chain, testis-specific-like
XP_025107285.1	tubulin alpha-8 chain-like
XP_025114702.1	LQP: peptidyl-prolyl cis-trans isomerase B-like
XP_025099135.1	LQP: arginine kinase-like
XP_025112799.1	endoplasmic reticulum chaperone BiP
XP_025081459.1	talin-1-like isoform X1
XP_025113391.1	LQP: spectrin alpha chain-like
XP_025093356.1	ATP synthase subunit beta, mitochondrial-like
XP_025091015.1	collagen alpha-5(VI) chain-like isoform X2
XP_025076319.1	phosphoenolpyruvate carboxykinase [GTP]-like
XP_025080404.1	calumenin-B-like isoform X1
XP_025078101.1	translation elongation factor 2-like
XP_025080019.1	leukocyte elastase inhibitor-like

XP_025104019.1	coiled-coil domain-containing protein 141-like isoform X1
XP_025076941.1	ATP synthase subunit alpha, mitochondrial-like
XP_025106309.1	gelsolin-like protein 2 isoform X2
XP_025095794.1	LQP: 60 kDa heat shock protein, mitochondrial-like
XP_025085337.1	heat shock protein HSP 90-alpha-like isoform X1
XP_025107756.1	endoplasmin-like
XP_025107717.1	rab GDP dissociation inhibitor alpha-like
XP_025083164.1	neprilysin-4-like
XP_025104607.1	histone H3-like
XP_025089563.1	clathrin heavy chain 1
XP_025110182.1	LQP: peroxidasin-like
XP_025092500.1	LQP: galectin-4-like
XP_025109457.1	ADP,ATP carrier protein-like
XP_025101259.1	serine/threonine-protein phosphatase 2A 65 kDa regulatory subunit A alpha isoform-like isoform X1
XP_025104976.1	histone H2B, gonadal
XP_025109714.1	LQP: glycogen debranching enzyme-like
XP_025115912.1	purine nucleoside phosphorylase-like
XP_025103811.1	glycogen phosphorylase, muscle form-like
XP_025077535.1	protein disulfide-isomerase A3-like
XP_025087821.1	1,4-alpha-glucan-branching enzyme-like isoform X1
XP_025081304.1	glyoxylate reductase/hydroxypyruvate reductase-like isoform X1
XP_025086690.1	myophilin-like
XP_025081483.1	uncharacterized protein LOC112556562
XP_025100353.1	calmodulin, striated muscle-like isoform X1
XP_025082249.1	glyoxylate reductase/hydroxypyruvate reductase-like
XP_025080083.1	retinal dehydrogenase 2-like
XP_025106061.1	LQP: UTP--glucose-1-phosphate uridylyltransferase-like
XP_025087407.1	phosphoglycerate kinase 1-like
XP_025095713.1	guanine nucleotide-binding protein subunit beta-2-like 1
XP_025094983.1	uncharacterized protein ZK1073.1-like isoform X1
XP_025088756.1	LQP: carbonyl reductase [NADPH] 1-like
XP_025097225.1	40S ribosomal protein S4-like
XP_025094855.1	LQP: aspartate aminotransferase, cytoplasmic-like
XP_025115754.1	aspartate aminotransferase, mitochondrial-like
XP_025088846.1	hsc70-interacting protein-like isoform X1
XP_025109846.1	40S ribosomal protein S7-like
XP_025112033.1	LQP: retrograde protein of 51 kDa-like
XP_025089532.1	60S acidic ribosomal protein P0-like
XP_025098733.1	chloride intracellular channel protein 4-like
XP_025090384.1	mediator of RNA polymerase II transcription subunit 15-like isoform X1
XP_025079145.1	eukaryotic initiation factor 4A-I-like isoform X1
XP_025082600.1	basement membrane-specific heparan sulfate proteoglycan core protein-like isoform X1
XP_025114842.1	uncharacterized protein LOC112576527 isoform X10
XP_025083191.1	peptidyl-prolyl cis-trans isomerase B-like isoform X1
XP_025089867.1	adenylyl cyclase-associated protein 1-like isoform X1
XP_025085574.1	60S ribosomal protein L9-like

XP_025083177.1	sorbitol dehydrogenase-like
XP_025097457.1	sorbitol dehydrogenase-like
XP_025103728.1	ras-related protein Rab-1A
XP_025112474.1	LQP: 40S ribosomal protein S5-like
XP_025080479.1	6-phosphogluconate dehydrogenase, decarboxylating-like
XP_025092026.1	protein disulfide-isomerase A6 homolog
XP_025105844.1	LQP: saccharopine dehydrogenase-like oxidoreductase
XP_025115350.1	glucose-6-phosphate isomerase-like isoform X1
XP_025085471.1	troponin I-like isoform X1
XP_025078437.1	40S ribosomal protein S6
XP_025098343.1	T-complex protein 1 subunit beta-like isoform X1
XP_025089361.1	isocitrate dehydrogenase [NADP], mitochondrial-like
XP_025088246.1	40S ribosomal protein S13
XP_025095375.1	uncharacterized protein LOC112564634
XP_025112159.1	cAMP-dependent protein kinase regulatory subunit isoform X1
XP_025109348.1	transitional endoplasmic reticulum ATPase
XP_025096543.1	ADP-ribosylation factor 2 isoform X1
XP_025115033.1	adenosylhomocysteinase-like
XP_025106186.1	40S ribosomal protein SA-like
XP_025088066.1	ubiquitin carboxyl-terminal hydrolase-like
XP_025105257.1	uncharacterized protein LOC112570831
XP_025087575.1	metalloproteinase inhibitor 3-like isoform X3
XP_025081444.1	60S ribosomal protein L23
XP_025096557.1	citrate synthase, mitochondrial-like
XP_025106401.1	uncharacterized protein LOC112571557 isoform X1
XP_025110318.1	12 kDa FK506-binding protein-like
XP_025076712.1	heterogeneous nuclear ribonucleoprotein 27C-like isoform X1
XP_025081291.1	arsenite methyltransferase-like
XP_025082245.1	LQP: myosin heavy chain, non-muscle-like
XP_025098518.1	stefin-C-like
XP_025086814.1	uncharacterized protein LOC112559692
XP_025102335.1	uncharacterized protein LOC112568962 isoform X1
XP_025113277.1	40S ribosomal protein S18
XP_025086205.1	nucleoside diphosphate kinase-like
XP_025086204.1	40S ribosomal protein S3
XP_025107794.1	ras-related protein Rap1
XP_025093088.1	60S ribosomal protein L6-like
XP_025083894.1	profilin-like
XP_025088543.1	uncharacterized protein LOC112560733
XP_025106310.1	gelsolin-like protein 2
XP_025086639.1	LQP: protein lethal(2)essential for life-like
XP_025091415.1	40S ribosomal protein S2
XP_025108453.1	ubiquitin-like modifier-activating enzyme 1
XP_025111655.1	spliceosome RNA helicase DDX39B
XP_025105436.1	calpain-B-like isoform X1
XP_025085932.1	plastin-1-like
XP_025087916.1	40S ribosomal protein S3a

XP_025090773.1	60S ribosomal protein L23a-like isoform X1
XP_025082063.1	tropomodulin-like
XP_025104850.1	elongation factor 1-gamma-like
XP_025107162.1	LQP: 60S ribosomal protein L3-like
XP_025109085.1	laminin subunit alpha-like
XP_025094847.1	glycine--tRNA ligase-like
XP_025096773.1	transaldolase-like isoform X1
XP_025112782.1	60S ribosomal protein L5-like
XP_025081510.1	pyruvate kinase PKM-like isoform X1
XP_025109667.1	polypyrimidine tract-binding protein 2-like isoform X1
XP_025088080.1	60S ribosomal protein L32-like
XP_025086455.1	dihydropyrimidinase-like
XP_025106938.1	mitochondrial-processing peptidase subunit beta-like
XP_025113992.1	quinone oxidoreductase-like isoform X1
XP_025085423.1	40S ribosomal protein S10-like
XP_025090121.1	60S acidic ribosomal protein P1-like
XP_025078139.1	60S ribosomal protein L17-like
XP_025087593.1	glycogen [starch] synthase-like
XP_025092427.1	flotillin-2-like
XP_025080098.1	60S ribosomal protein L8-like
XP_025105813.1	ribosome-binding protein 1-like isoform X1
XP_025081296.1	proteasome subunit alpha type-2
XP_025092955.1	aconitate hydratase, mitochondrial-like
XP_025103394.1	probable methylmalonate-semialdehyde dehydrogenase [acylating], mitochondrial
XP_025100959.1	uncharacterized protein LOC112568087
XP_025112729.1	uncharacterized protein LOC112575240
XP_025107611.1	laminin subunit beta-1-like
XP_025103993.1	titin homolog isoform X1
XP_025087837.1	60S ribosomal protein L12-like
XP_025116283.1	uncharacterized protein LOC112577430 isoform X1
XP_025096378.1	ras-like GTP-binding protein RHO
XP_025091729.1	60S ribosomal protein L19-like
XP_025096000.1	60S ribosomal protein L27-like
XP_025112013.1	40S ribosomal protein S9
XP_025094074.1	LQP: uncharacterized protein LOC112563873
XP_025092020.1	60S ribosomal protein L7-like
XP_025087859.1	LQP: BTB/POZ domain-containing protein KCTD12-like
XP_025103882.1	histone H2A
XP_025098510.1	40S ribosomal protein S15Aa
XP_025110976.1	phosphoglucomutase-1-like
XP_025097167.1	60S ribosomal protein L24-like
XP_025092850.1	dolichyl-diphosphooligosaccharide--protein glycosyltransferase subunit 2-like
XP_025115104.1	hemocyte protein-glutamine gamma-glutamyltransferase-like isoform X1
XP_025107641.1	universal stress protein A-like protein
XP_025105795.1	protein phosphatase 1B-like isoform X1
XP_025093952.1	uncharacterized protein LOC112563809
XP_025097674.1	peroxiredoxin-2-like

XP_025081438.1	LIM and SH3 domain protein F42H10.3-like isoform X1
XP_025086082.1	LQP: septin-11-like
XP_025083868.1	heterogeneous nuclear ribonucleoprotein D-like
XP_025100558.1	40S ribosomal protein S19-like isoform X1
XP_025097427.1	beta-parvin-like
XP_025106346.1	dolichyl-diphosphooligosaccharide--protein glycosyltransferase subunit 1-like
XP_025095580.1	chitotriosidase-1-like isoform X1
XP_025077177.1	60S ribosomal protein L4-like
XP_025085605.1	annexin A7-like
XP_025090122.1	60S ribosomal protein L30-like
XP_025076144.1	beta-1,3-glucan-binding protein-like
XP_025112876.1	uncharacterized protein LOC112575323
XP_025108539.1	ankyrin-2-like isoform X1
XP_025081749.1	guanine nucleotide-binding protein subunit beta isoform X2
XP_025082468.1	LQP: 60S ribosomal protein L18-like
XP_025096536.1	ubiquitin-conjugating enzyme E2 2
LQP= low quality protein	
