# Supplementary material for: Pomacea canaliculata Ampullar Proteome: A Nematode-Based Bio-Pesticide Induces Changes in Metabolic and Stress-Related Pathways
Source: Biology (Basel). 2021 Oct 15;10(10):1049. doi: 10.3390/biology10101049 (PMC8533556; doi:10.3390/biology10101049)
Supplement: Supplementary file 1 [file biology-10-01049-s001.zip › Table S3.pdf]

Table S3. List of common proteins identified in AmpC and AmpN. A label-free quantitative proteomic analysis was applied. For each protein it has been calculated the p-value and log_2 Fold change (AmpN/AmpC).

Accession	Description	P-value	log_2 Fold Change
XP_025089564.1	LQP: hemocyanin G-type, units Oda to Odg-like	3.11E-06	0.631516
XP_025089796.1	hemocyanin G-type, units Oda to Odg-like	0.000323	0.555688
XP_025106444.1	filamin-A-like isoform X1	0.000299	1.419625
XP_025112424.1	actin, adductor muscle	0.011005	0.83838
XP_025110090.1	actin, cytoplasmic	0.006853	0.513847
XP_025110638.1	LQP: myosin heavy chain, striated muscle-like	5.89E-06	5.289117
XP_025089549.1	cartilage matrix protein-like	0.329807	0.195573
XP_025102035.1	15-hydroxyprostaglandin dehydrogenase [NAD(+)]-like	0.015855	0.389144
XP_025078481.1	murinoglobulin-1-like isoform X8	0.046628	0.34875
XP_025086366.1	paramyosin-like isoform X1	6.29E-07	3.498598
XP_025093885.1	LQP: uncharacterized protein LOC112563776	4.27E-05	1.148421
XP_025103230.1	calponin-1-like	0.008385	-0.55247
XP_025083091.1	enolase-like	0.000142	0.558131
XP_025106551.1	collagen alpha-3(VI) chain-like isoform X18	0.00317	1.046923
XP_025106211.1	glutamate receptor 1-like	8.88E-06	1.742724
XP_025082312.1	glyceraldehyde-3-phosphate dehydrogenase-like isoform X1	0.010395	0.56158
XP_025104758.1	malate dehydrogenase, cytoplasmic-like	3.81E-05	2.592439
XP_025087209.1	transgelin-2-like	0.006039	0.834937
XP_025114701.1	peptidyl-prolyl cis-trans isomerase B-like	0.027084	0.723567
XP_025087809.1	vinculin-like isoform X1	0.000419	1.800653
XP_025099134.1	LQP: arginine kinase-like	0.000265	1.510341
XP_025083839.1	heat shock cognate 71 kDa protein	3.9E-05	2.888477
XP_025099490.1	heat shock protein 70 B2-like	0.006393	1.295692
XP_025103379.1	alpha-actinin, sarcomeric-like isoform X1	0.000361	4.139306
XP_025113955.1	glutathione S-transferase 1-like	0.000842	1.553176
XP_025113405.1	glutathione S-transferase S1-like	8.63E-05	1.972678
XP_025098567.1	myophilin-like	0.321925	-0.33801
XP_025095804.1	uncharacterized protein LOC112564910	0.403734	0.20436
XP_025114751.1	synaptic vesicle membrane protein VAT-1 homolog-like	0.009488	0.726411
XP_025104956.1	LQP: spectrin beta chain-like	0.000468	3.035385
XP_025090809.1	LQP: elongation factor 1-alpha-like	0.000708	3.831499
XP_025094704.1	protein disulfide-isomerase-like isoform X1	0.004507	1.359329
XP_025093079.1	14-3-3 protein epsilon-like isoform X1	0.037068	1.464123
XP_025090294.1	14-3-3 protein beta/alpha-A-like	0.005248	1.763492
XP_025080405.1	calumenin-like isoform X2	0.001715	-1.09339
XP_025078041.1	LQP: collagen alpha-5(VI) chain-like	0.418012	0.25486
XP_025106720.1	LQP: transketolase-like	0.000397	2.571769
XP_025109080.1	calreticulin-like	0.116147	0.145734
XP_025099080.1	alpha-crystallin A chain-like	0.026076	-0.19405
XP_025096926.1	protein lethal(2)essential for life-like	0.002006	-0.42869
XP_025077321.1	LQP: thrombospondin type-1 domain-containing protein 4-like	0.979517	0.002997
XP_025111140.1	far upstream element-binding protein 3-like	0.096432	-0.3441

XP_025110201.1	LQP: uncharacterized protein LOC112573811	5.36E-06	1.269061
XP_025080024.1	serine protease inhibitor 2.1-like isoform X1	0.008812	0.846907
XP_025080031.1	serine protease inhibitor 2.1-like	0.000502	0.958002
XP_025079300.1	myosin essential light chain, striated adductor muscle-like	0.731334	0.04562
XP_025082558.1	LQP: protein singed-like	0.022403	1.927912
XP_025090599.1	fructose-bisphosphate aldolase-like isoform X1	0.022681	1.050548
XP_025098992.1	superoxide dismutase [Cu-Zn]-like	0.951428	0.008747
XP_025083507.1	uncharacterized protein LOC112557719	0.237408	-0.32136
XP_025089044.1	malate dehydrogenase, mitochondrial-like isoform X1	0.000113	1.817043
XP_025086134.1	hemicentin-2-like isoform X1	0.000508	-0.95566
XP_025097192.1	NADP-dependent malic enzyme-like	0.267131	0.214143
XP_025084779.1	LQP: twitchin-like	0.609592	0.119425
XP_025108883.1	peptidyl-prolyl cis-trans isomerase-like	1.06E-06	1.797534
XP_025113499.1	PDZ and LIM domain protein 7-like isoform X1	0.005315	0.707244
XP_025089511.1	collagen alpha-6(VI) chain-like	0.000789	0.715515
XP_025086357.1	troponin T, skeletal muscle-like isoform X3	0.022699	0.539799
XP_025098387.1	catalase-like isoform X1	0.010952	2.622116
XP_025085843.1	dihydropyrimidinase-like isoform X1	0.000429	2.521828
XP_025084336.1	uncharacterized protein LOC112558220	0.046419	0.144776
XP_025107274.1	LQP: glutathione S-transferase Mu 2-like	0.000396	2.341715
XP_025089430.1	uncharacterized protein LOC112561269	0.007239	0.772014
XP_025107962.1	uncharacterized protein LOC112572474	0.003593	-0.36236
XP_025107262.1	uncharacterized protein LOC112572003	0.223464	0.986147
XP_025080714.1	2hiamine pyrophosphokinase 1-like isoform X1	0.475275	0.136653
XP_025092669.1	cartilage matrix protein-like	0.017131	-0.36215
XP_025087031.1	thymosin beta-like isoform X2	0.004896	-1.05945
XP_025078405.1	microtubule-associated protein futsch-like isoform X1	0.061955	-1.24907
XP_025078538.1	voltage-dependent anion-selective channel protein 2-like	0.012484	2.206812
XP_025100514.1	kinesin-like protein K39	0.003484	-1.03774
XP_025082853.1	uncharacterized protein LOC112557300	0.020629	-1.55671
XP_025104157.1	LQP: titin-like	0.000772	-1.64498
XP_025112526.1	rho GDP-dissociation inhibitor 1-like	0.435723	0.237909
XP_025087544.1	small heat shock protein p36-like	3.5E-06	-2.04098
XP_025099800.1	radixin-like	0.000492	1.316749
XP_025085606.1	uncharacterized protein LOC112559006	9.59E-05	-1.6973
XP_025091980.1	reticulon-1-like isoform X1	0.031882	0.663382
XP_025089524.1	collagen alpha-6(VI) chain-like	0.000468	1.516933
XP_025112685.1	LIM domain-containing protein WLIM2b-like	0.198805	-1.01772
XP_025092049.1	actin-depolymerizing factor 2-like	0.190762	-0.30598
XP_025081499.1	eukaryotic translation initiation factor 5A-1-like isoform X1	0.003639	-0.95098
XP_025094027.1	uncharacterized protein LOC112563852 isoform X1	0.019297	0.468742
XP_025105842.1	enolase-phosphatase E1-like	0.202763	0.465853
XP_025096983.1	myophilin-like	0.370785	0.495811
XP_025092675.1	60S acidic ribosomal protein P2-like	0.006832	2.005876
XP_025110989.1	xylose isomerase-like	0.01412	1.833387
XP_025082709.1	SH3 domain-binding glutamic acid-rich-like protein 3	0.01434	-0.79073
XP_025087966.1	copper transport protein ATOX1-like	0.00032	-0.77194
XP_025107348.1	superoxide dismutase [Cu-Zn]-like	0.048028	0.14353

XP_025085667.1	lysosomal aspartic protease-like	0.024059	2.32086
XP_025104087.1	titin-like isoform X1	0.000689	-2.06979
XP_025078628.1	LQP: neurofilament medium polypeptide-like	0.025379	1.346143
XP_025106788.1	uncharacterized protein LOC112571747	0.726597	0.197216
XP_025109789.1	transforming growth factor-beta-induced protein ig-h3-like	0.033214	-1.188
XP_025096878.1	uncharacterized protein LOC112565575 isoform X1	0.002002	-1.32377
XP_025082706.1	protein/nucleic acid deglycase DJ-1-like	0.002122	2.024145
XP_025089277.1	cofilin-like	0.079226	0.685441
XP_025098601.1	troponin C-like isoform X1	0.002188	-1.41944
XP_025106684.1	uncharacterized protein LOC112571691	0.13513	-0.34197
XP_025090849.1	vegetative incompatibility protein HET-E-1-like	0.001314	1.324563
XP_025104082.1	barrier-to-autointegration factor-like	0.047906	0.14353
XP_025093398.1	actin-interacting protein 1-like	0.003751	1.623441
XP_025095112.1	elongation factor 1-beta-like	0.027983	1.365221
XP_025082954.1	uncharacterized protein LOC112557363	0.356392	-1.03829
XP_025077458.1	LQP: fibrillin-2-like	0.386627	3.635899
XP_025081260.1	collagen alpha-1(I) chain-like	0.003941	-1.51975
XP_025099638.1	ganglioside GM2 activator-like	0.03827	0.151989
XP_025081261.1	collagen alpha-1(I) chain-like isoform X1	0.855228	-0.05926
XP_025104800.1	uncharacterized protein LOC112570531	0.010964	-0.85766
XP_025104799.1	uncharacterized protein LOC112570529	0.001003	-1.72428
XP_025113673.1	fatty acid-binding protein, liver-like	0.132365	-0.65129
XP_025084055.1	uncharacterized protein LOC112558076	0.064938	0.669395
XP_025090775.1	LQP: tensin-1-like	5.75E-06	-1.77371
XP_025083419.1	small cardioactive peptides-like isoform X1	0.002106	-1.29382
XP_025083634.1	thioredoxin-1-like	0.034374	-1.58664
XP_025094101.1	FK506-binding protein 2-like	0.002086	-1.02828
XP_025111371.1	LQP: 40S ribosomal protein S12-like	6.42E-05	-1.38759
XP_025112868.1	uncharacterized protein LOC112575321 isoform X1	0.020812	1.695959
XP_025092572.1	von Willebrand factor D and EGF domain-containing protein-like	0.790373	0.251846
XP_025109048.1	peroxiredoxin-like isoform X1	0.893765	-0.05183
XP_025114384.1	PDZ and LIM domain protein 3-like isoform X1	0.000647	-1.60308
XP_025077943.1	LQP: cartilage matrix protein-like	0.127128	-1.12619
XP_025105802.1	lactoylglutathione lyase-like isoform X1	0.218581	0.426436
XP_025104144.1	mammalian ependymin-related protein 1-like	0.000133	-1.04629
XP_025076135.1	fructose-1,6-bisphosphatase 1-like	0.234194	1.070642
XP_025082862.1	PDZ and LIM domain protein 5-like	0.000199	-2.36176
XP_025082818.1	triosephosphate isomerase-like	0.137427	1.041259
XP_025116005.1	uncharacterized protein LOC112577210 isoform X1	0.055437	-0.59311
XP_025089133.1	fatty acid-binding protein, adipocyte-like	0.000652	-0.61427
XP_025087122.1	myotrophin-like	0.029611	-0.86316
XP_025101599.1	phospholipid transfer protein C2CD2L-like	0.133925	-0.96324
XP_025078843.1	glycogenin-1-like isoform X1	0.00642	-1.0357
XP_025082010.1	myosin-2 essential light chain-like	0.035777	-3.49412
LQP= low quality protein			
